# Colorectal Cancer-Associated Microbiome Remodeling Implicates Host Microbial Metabolite–Receptor Axes

**DOI:** 10.3390/ph19101608

**Published:** 2026-10-10

**Authors:** Michail Spathakis, George Kolios, Vasilis Paspaliaris, Nikolas Dovrolis

**Affiliations:** 1 Laboratory of Pharmacology, Faculty of Medicine, Democritus University of Thrace, 68100 Alexandroupolis, Greece; michael.spathakis@outlook.com (M.S.); gkolios@med.duth.gr (G.K.); 2 Paspa Pharmaceuticals Pty Ltd., Caulfield, Melbourne 3162, Australia; 3 Biogut Technologies Inc., Fort Worth, TX 76104, USA; 4 Laboratory of Biology, Faculty of Medicine, Democritus University of Thrace, 68100 Alexandroupolis, Greece

**Keywords:** colorectal cancer, adenoma, microbiome, metabolite, host receptor

## Abstract

**Background/Objectives**: Colorectal neoplasia is accompanied by reproducible changes in the gut microbiome and fecal metabolome, but the receptor systems through which altered microbial metabolic outputs may interface with the host remain incompletely defined. We investigated whether disease-associated microbial functional remodeling converges on metabolite–sensing receptor axes that could prioritize mechanistic and pharmacological hypotheses across the adenoma-to-carcinoma sequence. **Methods**: We analyzed 1534 publicly available stool shotgun metagenomes from 11 colorectal neoplasia cohorts, including 624 controls, 209 adenomas and 701 colorectal cancers (CRCs). Study-adjusted species and unstratified MetaCyc pathway models were combined with stage-pattern classification and mapping of microbial pathways to predefined metabolite–receptor axes. Findings were examined in two independent fecal metabolomics datasets. **Results**: CRC was associated with 68 species and 249 unstratified pathways at false discovery rate q < 0.05. Among CRC-associated features, 17 species and 36 pathways were adenoma-concordant, whereas 37 species and 170 pathways were CRC-specific. Of 136 pathways mapped to seven receptor-linked ligand classes, 79 were FDR-supported. Nucleotide/purinergic metabolism contained 39 FDR-supported pathways, predominantly lower in CRC, whereas fatty-acid/short-chain fatty acid signaling contained 21 FDR-supported pathways, predominantly higher in CRC. Independent fecal metabolomics showed the strongest axis-level convergence for these two axes. **Conclusions**: Colorectal neoplasia is associated with reproducible microbial functional remodeling that can be organized into biologically defined metabolite–receptor axes. Fatty-acid/SCFA and nucleotide/purinergic pathways emerge as the leading candidates for prospective functional and pharmacological investigation. The analysis prioritizes testable host–microbiome signaling modules but does not establish metabolite flux, receptor activation or therapeutic efficacy.

## 1. Introduction

Colorectal cancer (CRC) develops within a metabolically active mucosal ecosystem in which the intestinal epithelium, immune and stromal compartments, diet-derived substrates and the gut microbiota interact across the adenoma-to-carcinoma sequence. Although screening, prevention and treatment have improved substantially, the biological links between the intestinal environment and colorectal carcinogenesis remain incompletely understood. The multistep nature of CRC, from normal mucosa to adenoma and invasive carcinoma, creates an opportunity to ask whether microbial alterations are already detectable before cancer and whether these alterations become reorganized as neoplasia progresses.

Over the past decade, shotgun metagenomic studies have shown a direct association of CRC with reproducible alterations in stool microbial composition. Multiple independent cohorts and cross-cohort analyses have reported enrichment of oral-associated anaerobes and pathobionts, alongside depletion of commensal taxa [1,2,3,4]. These studies established that the CRC stool microbiome has a recurrent disease-associated signature. At the same time, they also showed that this signature is not a single-microbe phenomenon, but a distributed pattern involving multiple taxa, ecological states and functional pathways. Several CRC-associated taxa also have experimentally supported links to epithelial signaling, immune modulation and genotoxicity, including *Fusobacterium nucleatum*, enterotoxigenic *Bacteroides fragilis* and pks-positive *Escherichia coli* [5,6,7,8]. These organism-specific mechanisms establish that microbiome alterations can influence colorectal tumor biology, but they do not explain how broader multi-cohort functional pathway changes should be interpreted at the host–microbe interface.

Microbial metabolism provides one way to connect stool metagenomic changes with host biology. Gut microbial metabolites are not only metabolic end products; many also act as signaling molecules that can influence epithelial cells, immune cells, stromal cells, vascular cells and enteric neuronal circuits. Fecal metagenomic and metabolomic studies have shown that colorectal neoplasia is accompanied by changes in microbial functions and measured metabolites from early lesions to invasive cancer. Yachida et al. reported stage-specific metagenomic and metabolomic changes across adenoma, intramucosal carcinoma and more advanced CRC [3]. Sinha et al. showed that fecal metabolites mediated some CRC associations with microbial taxa, including *Fusobacterium* and *Porphyromonas* [9]. Kim et al. reported fecal metabolomic signatures in colorectal adenoma associated with gut microbiota and early events of CRC pathogenesis [10]. More recently, metabolomic analyses across normal, adenoma and CRC groups identified progression-associated metabolites, including oleic acid and allocholic acid, with functional effects in CRC models [11]. A prospective post-adenoma study further reported persistent gut microbiome and fecal metabolome differences many years after adenoma resection, supporting the possibility that colorectal neoplasia is associated with durable microbial-metabolic states rather than only transient changes around an active lesion [12].

Despite these advances, most CRC microbiome studies still focus mainly on which organisms, genes or pathways differ between disease groups. This remains necessary, but it leaves an important biological question unresolved: microbial metabolites matter because host cells can sense them. Short-chain fatty acids, lactate, nucleotides, amino-acid derivatives, indoles, histamine, catecholamine-related compounds and lipid mediators can engage host receptor systems expressed across epithelial, immune, stromal, vascular and neuronal compartments. Previous work from our group highlighted microbial metabolite receptors as a host-side layer of host–microbe communication in cancer, including altered expression of several receptor systems in colorectal tumors [13]. The unresolved question is therefore not whether microbial pathways can produce metabolites, but whether CRC-associated microbial pathway remodeling and CRC-associated host receptor remodeling point toward shared ligand-sensing axes.

This question is particularly relevant for fatty-acid, short-chain fatty-acid and lactate sensing, and for nucleotide and purinergic biology. Butyrate and other short-chain fatty acids can influence colonocyte metabolism, histone acetylation, inflammation and tumor cell behavior in context-dependent ways [14,15,16,17]. Lactate sensing through HCAR1 has also been shown to affect chemokine production and recruitment of immunosuppressive myeloid cells in colorectal tumors [17]. In parallel, microbial nucleotide metabolism has been linked to treatment response biology in rectal cancer, where microbiota-mediated nucleotide biosynthesis was associated with resistance to neoadjuvant chemoradiotherapy [18]. These studies do not imply that metagenomic pathway abundance is equivalent to metabolite flux or receptor activation. They do show that microbial ligand classes can intersect with receptor-mediated processes relevant to colorectal neoplasia.

In this study, we performed a large-scale integrated analysis of 1534 stool shotgun metagenomes from 11 colorectal neoplasia cohorts contained within publicly available metagenomic datasets [19] together with two independent fecal metabolomics resources. In our analysis, following a different level of interpretation for biomarker discovery, we aimed to connect disease-associated metagenomic functional shifts to ligand classes that can plausibly engage several metabolite-sensing receptor systems in colorectal neoplasia. With this approach, we developed a receptor-informed framework for organizing microbiome-associated functional changes across colorectal neoplasia and prioritizing specific host–microbiome axes for prospective mechanistic testing.

## 2. Results

### 2.1. Study Overview and Analytical Framework

The final analysis set comprised 1534 stool metagenomes from 11 curatedMetagenomicData studies. The cohort included 624 controls, 209 adenoma samples and 701 CRC samples. The following five studies contributed adenoma samples: YachidaS_2019 [3], ZellerG_2014 [4], FengQ_2015 [20], ThomasAM_2018a [1] and HanniganGD_2017 [21]. YachidaS_2019 [3] was the largest contributor with 571 samples. ZellerG_2014 [4] contributed 156 samples, WirbelJ_2018 [2] contributed 125, YuJ_2015 [22] contributed 112, VogtmannE_2016 [23] contributed 110, FengQ_2015 [20] contributed 109, HanniganGD_2017 [21] contributed 81, ThomasAM_2019_c [1] contributed 80, ThomasAM_2018a [1] contributed 70, and GuptaA_2019 [24] and ThomasAM_2018b [1] contributed 60 each (Table 1).

**Figure 1:**
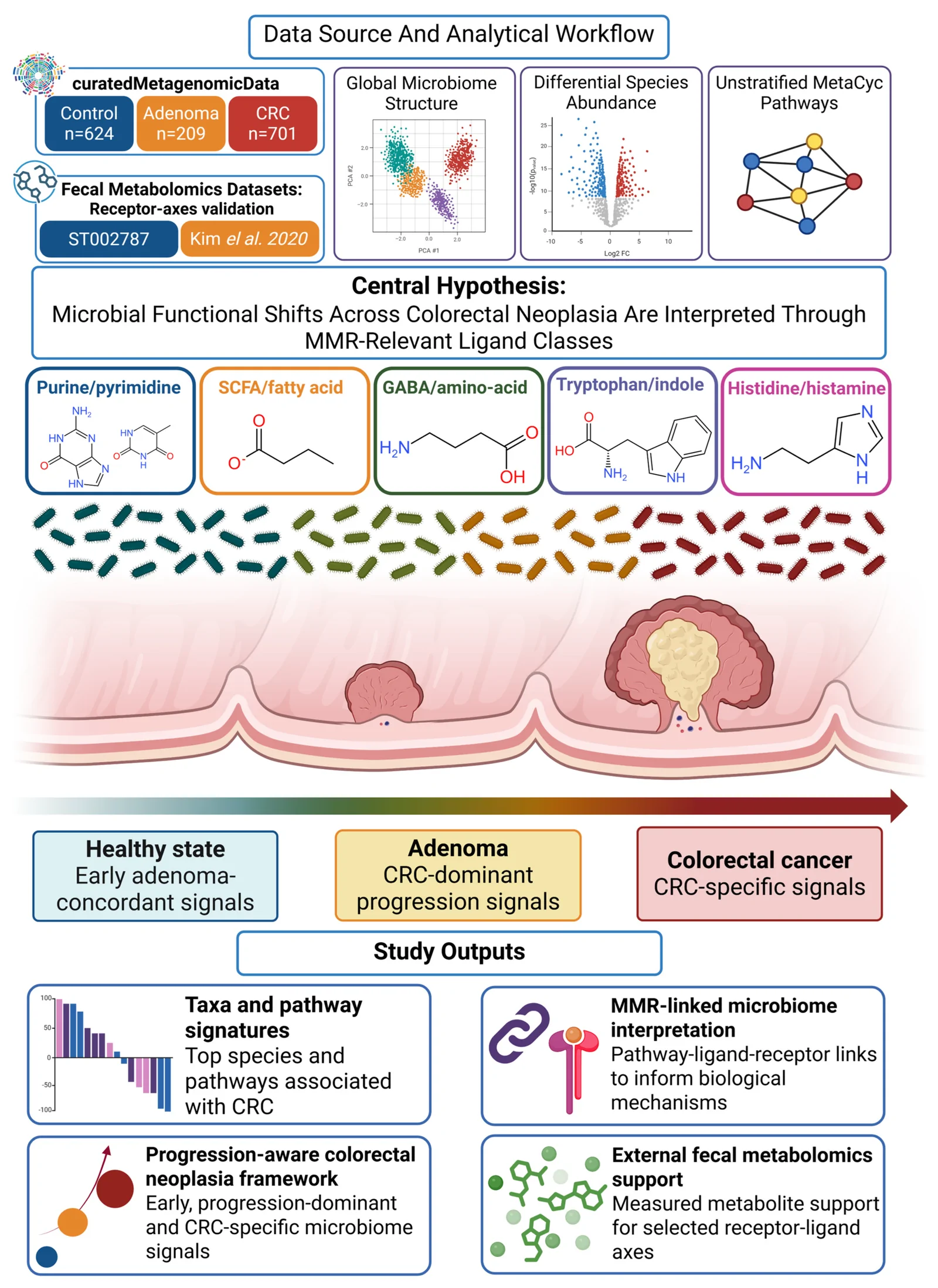
Study overview and integrated analytical framework. Public stool metagenomes from curatedMetagenomicData were analyzed across control, adenoma and CRC groups. The final metagenomic analysis set included 1534 samples from 11 studies. Species abundance and unstratified MetaCyc pathway abundance profiles were prevalence-filtered, CLR-transformed and analyzed using study-adjusted models. The workflow assessed global microbiome structure, differential species and pathway associations, adenoma-CRC concordance, MMR ligand-class mapping and external fecal metabolomics support. Created in BioRender. Spathakis, M. (2026) https://BioRender.com/cq940ss (accessed on 1 October 2026).

After filtering, 217 species-level features and 420 unstratified MetaCyc pathways were retained for the main taxonomic and functional analyses. Figure 1 summarizes the analysis workflow, including study selection, species and pathway feature extraction, CLR transformation, study-adjusted modeling, adenoma-CRC concordance analysis, MMR ligand-class mapping and external fecal metabolomics support.

### 2.2. Global Microbiome Structure Across Colorectal Neoplasia

Species-level alpha-diversity distributions showed modest group differences across controls, adenoma and CRC. Observed species richness was 79.4 ± 23.7 in controls, 78.5 ± 27.2 in adenoma and 86.0 ± 25.9 in CRC. Shannon diversity was 2.707 ± 0.577, 2.676 ± 0.571 and 2.781 ± 0.569, respectively. Simpson diversity was 0.850 ± 0.130, 0.850 ± 0.125 and 0.860 ± 0.123, respectively.

In study-adjusted ANOVA models, disease_group was associated with observed species richness (F = 26.473, df = 2, and *p* = 4.99 × 10^−12^) and Shannon diversity (F = 4.996, df = 2, and *p* = 0.007). Simpson diversity did not differ significantly by disease_group after study adjustment (F = 1.295, df = 2, and *p* = 0.274). These tests indicated modest disease-associated differences in richness and Shannon diversity rather than large alpha-diversity separation among groups.

**Figure 2:**
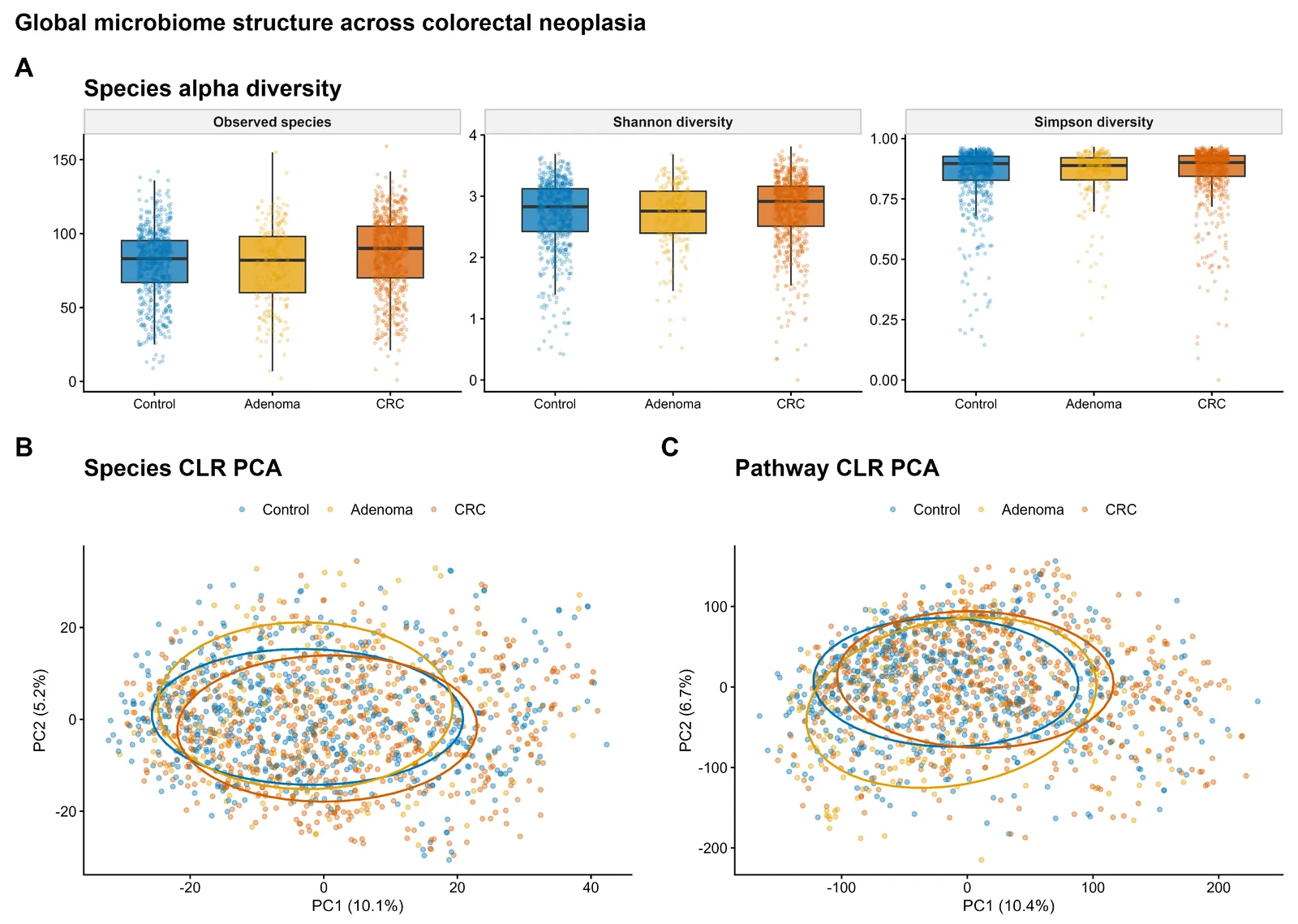
Global microbiome structure across colorectal neoplasia. (**A**) Species-level alpha diversity across Control, Adenoma and CRC groups, assessed using observed species richness, Shannon diversity and Simpson diversity. Each point represents an individual sample; boxplots summarize the median and interquartile range. (**B**) Principal component analysis (PCA) of centered log-ratio (CLR)-transformed species abundance profiles. (**C**) PCA of CLR-transformed unstratified MetaCyc pathway abundance profiles. Blue, yellow and orange represent Control, Adenoma and CRC samples, respectively, across all panels. In panels (**B**,**C**), each point represents an individual sample, ellipses summarize group dispersion in PCA space, and percentages on the axes indicate the variance explained by each principal component.

CLR principal component analysis showed overlapping disease-group distributions in both feature layers. For species CLR profiles, PC1 explained 10.1% and PC2 explained 5.2% of variance. For pathway CLR profiles, PC1 explained 10.4% and PC2 explained 6.7% of variance. In study-adjusted PERMANOVA, disease_group was associated with both species and pathway CLR profiles. For species profiles, disease_group explained R^2^ = 0.0054 after study adjustment (F = 4.508, *p* = 0.001). For pathway profiles, disease_group explained R^2^ = 0.0048 (F = 4.033, *p* = 0.001). Study_name explained a larger fraction of variance than disease_group in both layers (Figure 2). The same CLR PCA coordinates are additionally displayed by source study in Supplementary Figure S3, illustrating the substantial between-study structure present in both feature layers.

### 2.3. CRC-Associated Microbial Species and Unstratified Microbial Pathways

**Figure 3:**
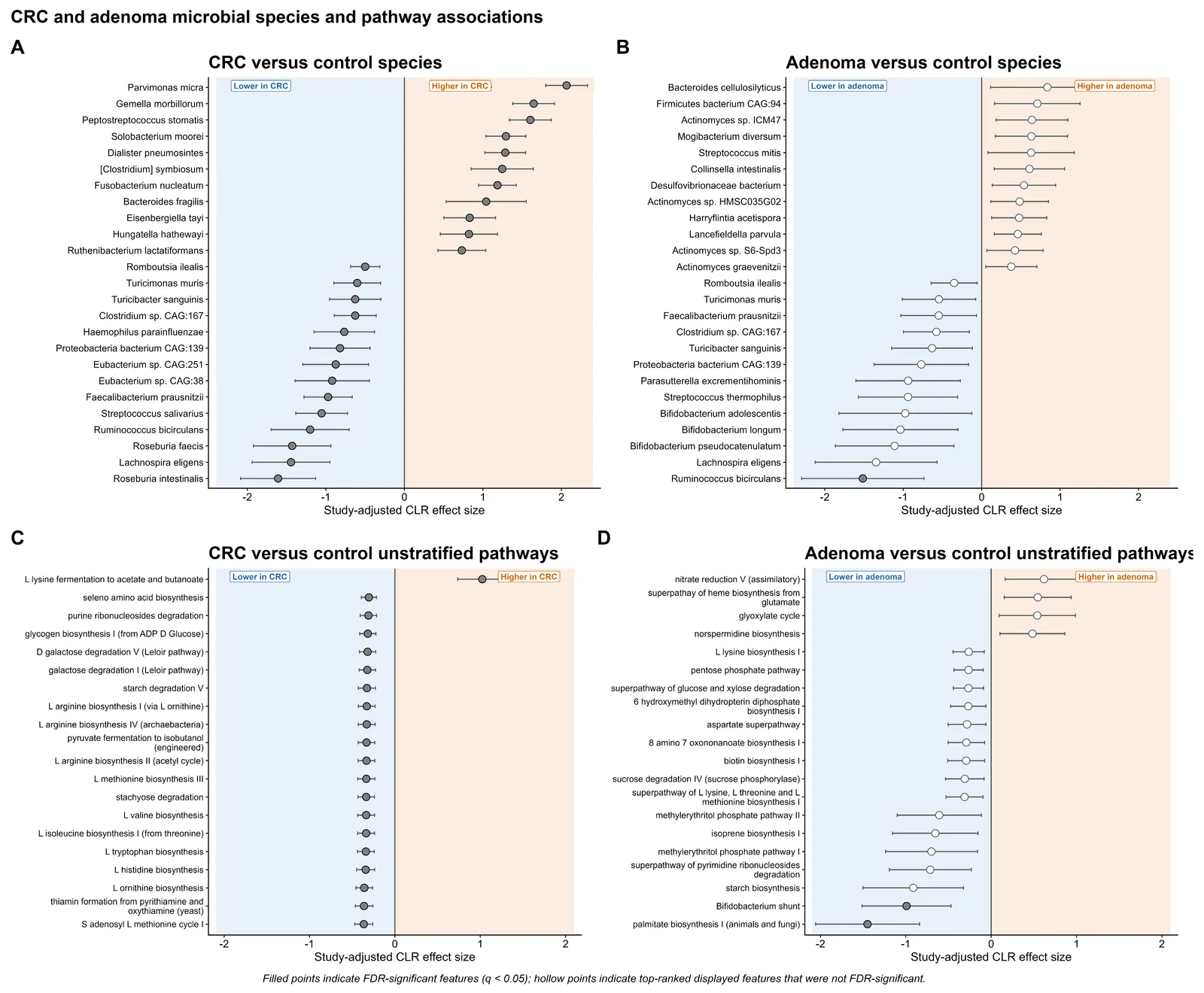
CRC- and adenoma-associated microbial species and unstratified microbial pathways. (**A**) Selected species in CRC versus Control. (**B**) Selected species in Adenoma versus Control. (**C**) Selected unstratified MetaCyc pathways in CRC versus Control. (**D**) Selected unstratified MetaCyc pathways in Adenoma versus Control. Points show study-adjusted CLR effect sizes with approximate 95% confidence intervals. The displayed features are selected for visualization and do not represent all tested or significant features. Filled points indicate FDR-significant features (q < 0.05), whereas hollow points indicate top-ranked displayed features that did not reach FDR significance. Shaded regions indicate the direction of the association relative to controls. Effects represent associations in metagenomic species abundance or pathway potential, not direct measurements of microbial activity or metabolite concentrations.

Study-adjusted differential modeling showed a broader microbial signal in CRC than in adenoma. In the CRC-versus-Control comparison, 68 of 217 tested species were FDR-significant at q < 0.05. Twenty-seven species were higher in CRC and 41 were lower in CRC. The smallest species-level q-value was 1.99 × 10^−35^. Species higher in CRC included *Parvimonas micra*, *Gemella morbillorum*, *Peptostreptococcus stomatis* and *Fusobacterium nucleatum*. Species lower in CRC included *Roseburia intestinalis*, *Lachnospira eligens*, *Roseburia faecis* and *Faecalibacterium prausnitzii*. Figure 3A displays a selected subset of the CRC-associated species for readability and does not show all 68 FDR-significant species.

The adenoma-versus-Control species signal was limited. One of 217 tested species, *Ruminococcus bicirculans*, was FDR-significant at q < 0.05 and was lower in adenoma than in controls (q = 0.0320). Figure 3B displays the FDR-significant adenoma-associated species together with selected top-ranked species that did not reach FDR significance, to show the broader effect-size distribution without implying statistical significance for all displayed features.

At the unstratified MetaCyc pathway level, CRC-versus-Control modeling identified 249 FDR-significant pathways among 420 tested pathways. Eighty-seven pathways were higher in CRC and 162 were lower in CRC. The smallest pathway-level q-value was 5.99 × 10^−10^. Higher-in-CRC pathways included L-lysine fermentation to acetate and butanoate, the superpathway of fatty acid biosynthesis and the superpathway of L-tryptophan biosynthesis. Lower-in-CRC pathways included S-adenosyl-L-methionine cycle I, L-ornithine biosynthesis, L-histidine biosynthesis, L-tryptophan biosynthesis and purine ribonucleosides degradation. Figure 3C displays a selected subset of the CRC-associated pathways for readability and does not show all 249 FDR-significant pathways.

In adenoma, 2 of 420 tested unstratified pathways were FDR-significant at q < 0.05, and both were lower in adenoma than in controls. These were palmitate biosynthesis I and the Bifidobacterium shunt. Figure 3D displays these FDR-significant pathways together with selected top-ranked adenoma-associated pathways that did not reach FDR significance. These effects describe differential metagenomic species abundance or pathway abundance, not measured microbial activity, pathway flux or metabolite concentration.

### 2.4. CRC-Associated Microbial Pathways Map Quantitatively to MMR-Relevant Ligand Classes

The MMR analysis mapped 136 CRC-versus-Control unstratified MetaCyc pathways to seven receptor-relevant ligand classes. Seventy-nine mapped pathways were FDR-significant at q < 0.05. The largest axis was nucleotide/purinergic metabolism, with 64 mapped pathways and 39 FDR-supported pathways. Most FDR-supported pathways in this class were lower in CRC (36 lower, three higher), with a median significant CLR effect of −0.230 and a minimum q-value of 6.40 × 10^−9^. This axis was annotated to ADORA, P2RY and P2RX receptor systems.

Fatty-acid/SCFA signaling contained 44 mapped pathways and 21 FDR-supported pathways. The FDR-supported direction was predominantly higher in CRC (16 higher, five lower), with a median significant CLR effect of 0.423 and a minimum q-value of 1.21 × 10^−9^. This axis was annotated to FFAR, HCAR, GPR84, GPR119 and PPAR-related systems. The presence of both higher- and lower-in-CRC pathways indicates that this ligand class is internally heterogeneous; the positive median therefore summarizes the predominant direction of the mapped pathway set and should not be interpreted as uniform activation or suppression of fatty-acid/SCFA metabolism.

**Figure 4:**
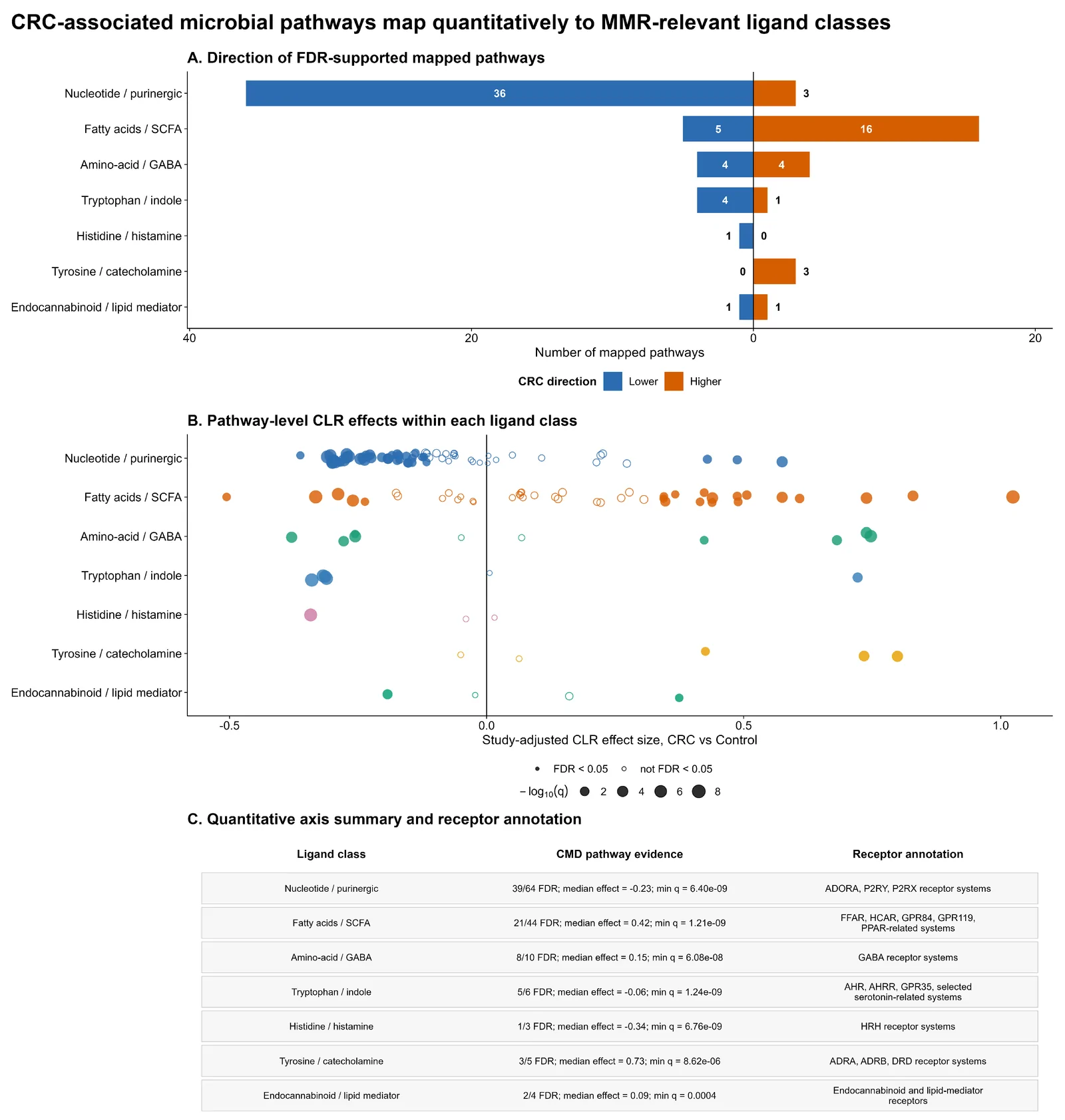
CRC-associated microbial pathways map quantitatively to MMR-relevant ligand classes. Panel (**A**) shows the direction of FDR-supported mapped pathways for the CRC-versus-Control contrast. Bars extending left indicate pathways lower in CRC, and bars extending right indicate pathways higher in CRC. Panel (**B**) shows pathway-level CLR effects within each ligand class, with point size proportional to −log10(q) and point shape indicating FDR support. Panel (**C**) summarizes axis-level pathway evidence and receptor annotations. The receptor annotations provide biological context for metagenomic pathway shifts and do not imply measured receptor expression or receptor activation. Axis-level summaries describe the predominant distribution of mapped pathway effects and do not imply that all pathways within a ligand class change in the same direction.

The remaining axes were smaller but contributed receptor-relevant pathway structure. Amino-acid/GABA contained 10 mapped pathways and eight FDR-supported pathways, with balanced higher and lower directions. Tryptophan/indole contained six mapped pathways and five FDR-supported pathways, most of which were lower in CRC. Histidine/histamine contained three mapped pathways and one FDR-supported pathway, which was lower in CRC. Tyrosine/catecholamine contained five mapped pathways and three FDR-supported pathways, all higher in CRC. The endocannabinoid/lipid-mediator axis contained four mapped pathways and two FDR-supported pathways, with one higher and one lower in CRC (Figure 4).

Representative pathway examples made the axis-level mapping biologically interpretable. Nucleotide/purinergic mappings included purine ribonucleosides degradation and adenosine ribonucleotides de novo biosynthesis. Fatty-acid/SCFA mappings included L-lysine fermentation to acetate and butanoate and pyruvate fermentation to acetate and lactate II. Tryptophan/indole mappings included L-tryptophan biosynthesis and the superpathway of L-tryptophan biosynthesis. Amino-acid/GABA mappings included the GABA shunt and L-glutamate degradation VIII, whereas histidine/histamine mappings included L-histidine biosynthesis.

Pathway-level effects showed that the MMR mapping was not driven only by pathway counts and also revealed substantial within-axis heterogeneity. The nucleotide/purinergic axis showed a comparatively coherent negative pattern, with 36 of 39 FDR-supported pathways lower in CRC, whereas the fatty-acid/SCFA axis contained both higher- and lower-in-CRC pathways despite an overall predominance of positive effects. Accordingly, axis-level summaries are used to organize related biochemical functions, whereas the direction and identity of individual pathways remain necessary for mechanistic interpretation. The receptor annotations in Figure 4 are therefore used as biological context for quantified metagenomic pathway effects.

These receptor annotations were not used to infer receptor expression, receptor occupancy or receptor activation in the metagenomic cohorts. They were used to determine whether CRC-associated microbial pathway shifts mapped to ligand classes for which colorectal tumors have previously shown altered microbial metabolite receptor expression. Under this interpretation layer, the clearest convergence axes were fatty-acid/SCFA-lactate biology and nucleotide/purinergic metabolism. The former combined predominantly higher CRC-associated microbial pathway effects with receptor families involved in fatty-acid, SCFA and lactate sensing, whereas the latter combined a larger set of predominantly lower CRC-associated nucleotide-related pathways with purinergic receptor annotations. The remaining mapped axes were retained as receptor-relevant pathway signals but were smaller or more mixed in direction.

### 2.5. Adenoma-CRC Concordance and Stage-Associated Microbial Signatures

**Figure 5:**
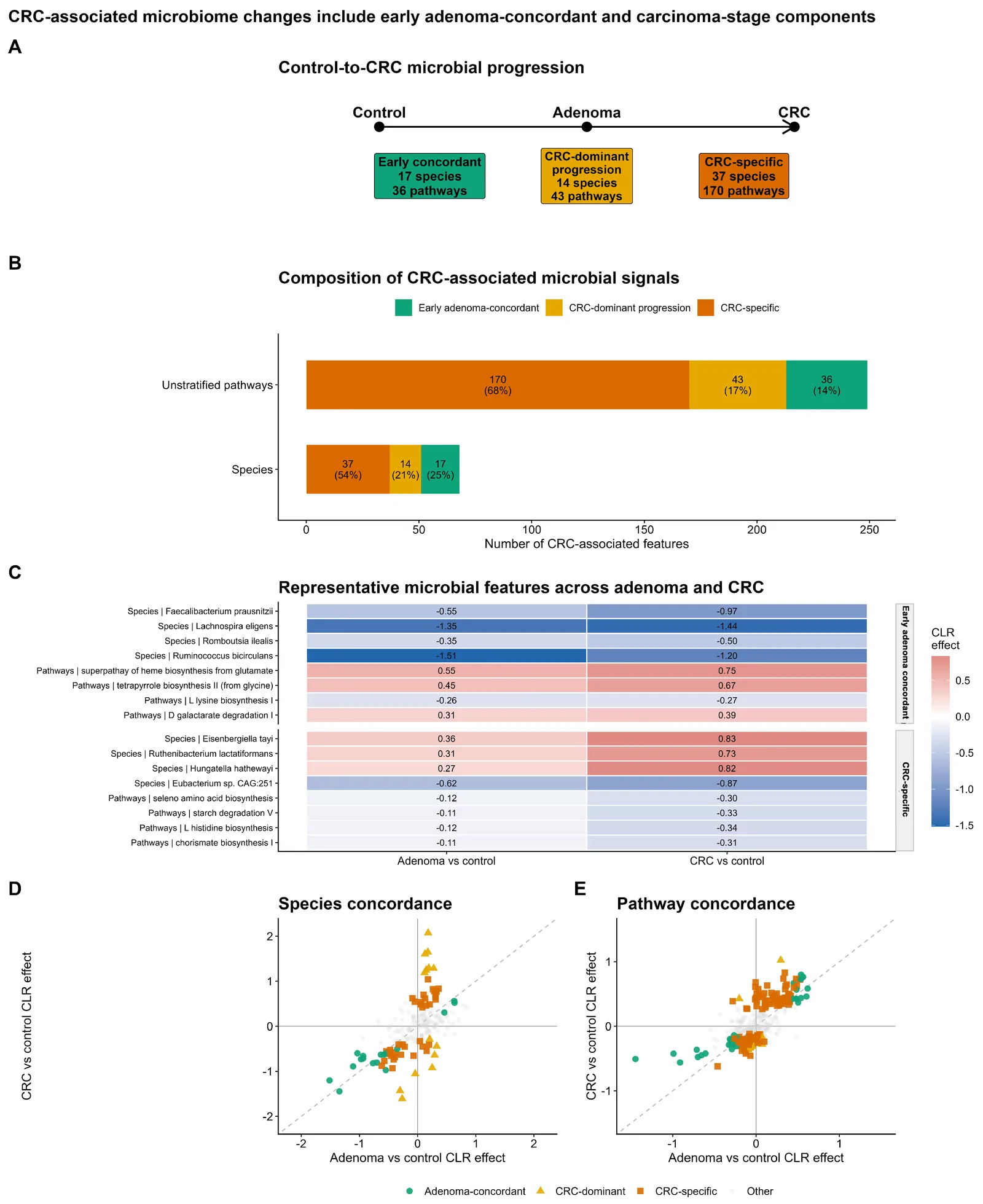
Adenoma-CRC concordance distinguishes early neoplasia and CRC-stage microbial signals. Panel (**A**) shows the partitioning of CRC-associated microbial signals across the control-to-adenoma-to-CRC sequence. Panel (**B**) quantifies the composition of CRC-associated species and unstratified pathways across the three feature classes. Panel (**C**) shows representative microbial species and pathways across adenoma and CRC, with tile color indicating the study-adjusted CLR effect size relative to controls. Panels (**D**,**E**) show adenoma-versus-Control and CRC-versus-Control effect concordance for species and pathways, respectively.

The classification separated CRC-associated features into adenoma-concordant CRC signals, CRC-dominant signals and CRC-specific signals. Among 68 CRC-associated species, 17 were classified as adenoma-concordant CRC signals (25%), 14 as CRC-dominant signals (21%) and 37 as CRC-specific signals (54%). Among 249 CRC-associated unstratified pathways, 36 were adenoma-concordant CRC signals (14%), 43 were CRC-dominant signals (17%) and 170 were CRC-specific signals (68%) (Figure 5A,B).

At the full tested-feature level, one of 217 species and two of 420 unstratified pathways were FDR-significant in Adenoma versus Control models. By contrast, 68 species and 249 unstratified pathways were FDR-significant in CRC versus Control models. The ordinal Control-to-Adenoma-to-CRC model identified 68 species and 248 unstratified pathways at q < 0.05. Representative species and pathways from these stage-pattern classes showed distinct adenoma and CRC effect patterns when visualized by study-adjusted CLR effect size (Figure 5C). Accordingly, the 17 species and 36 pathways classified as adenoma-concordant represent CRC-associated features with concordant nominal evidence in adenoma and should not be interpreted as FDR-significant adenoma associations.

The concordance plots separated features already directionally shifted in adenoma from features that were primarily shifted in CRC (Figure 5D,E). This classification provides a stage-aware summary of cross-sectional metagenomic association patterns. It does not establish temporal causality or individual-level adenoma-to-carcinoma progression.

### 2.6. Independent Fecal Metabolomics Convergence with MMR-Relevant Axes

We next assessed whether the pathway-derived MMR axes showed biological-axis-level convergence with independently measured fecal metabolites. ST002787 included 102 samples after import, with 30 controls, 37 adenoma samples and 35 CRC samples. The imported feature matrices contained 1074 positive ion-mode features and 567 negative ion-mode features before result-level harmonization. Because the metabolomics and metagenomic cohorts were independent, the analysis compared biological-axis summaries rather than paired sample-level microbiome–metabolite associations.

For the CRC-versus-Control contrast, the strongest concordant signals involved fatty-acid/SCFA and nucleotide/purinergic axes. Fatty-acid/SCFA signaling had 24 measured ST002787 metabolites, 10 FDR-supported metabolites and a positive median significant metabolite log2 fold-change of 2.043. The corresponding metagenomic axis had 44 mapped pathways, 21 FDR-supported pathways and a positive median significant CLR effect of 0.423. Nucleotide/purinergic metabolism had 68 measured metabolites, five FDR-supported metabolites and a negative median significant metabolite log2 fold-change of −1.815. The corresponding metagenomic axis had 64 mapped pathways, 39 FDR-supported pathways and a negative median significant CLR effect of −0.230. The ST002787 findings were unchanged in missing-value sensitivity analyses. Because the retained analyte matrices contained no missing abundance observations requiring replacement, available-case analysis reproduced the original differential results, and all 10 FDR-supported metabolites assigned to the broad fatty-acid/SCFA category retained the same effect direction and q < 0.05 under the sensitivity specifications. These FDR-supported metabolites were fatty-acid and oxylipin species rather than canonical short-chain fatty acids such as acetate, propionate or butyrate; the metabolomic convergence should therefore be interpreted at the broader fatty-acid-related axis level rather than as direct evidence of altered canonical SCFA concentrations.

**Figure 6:**
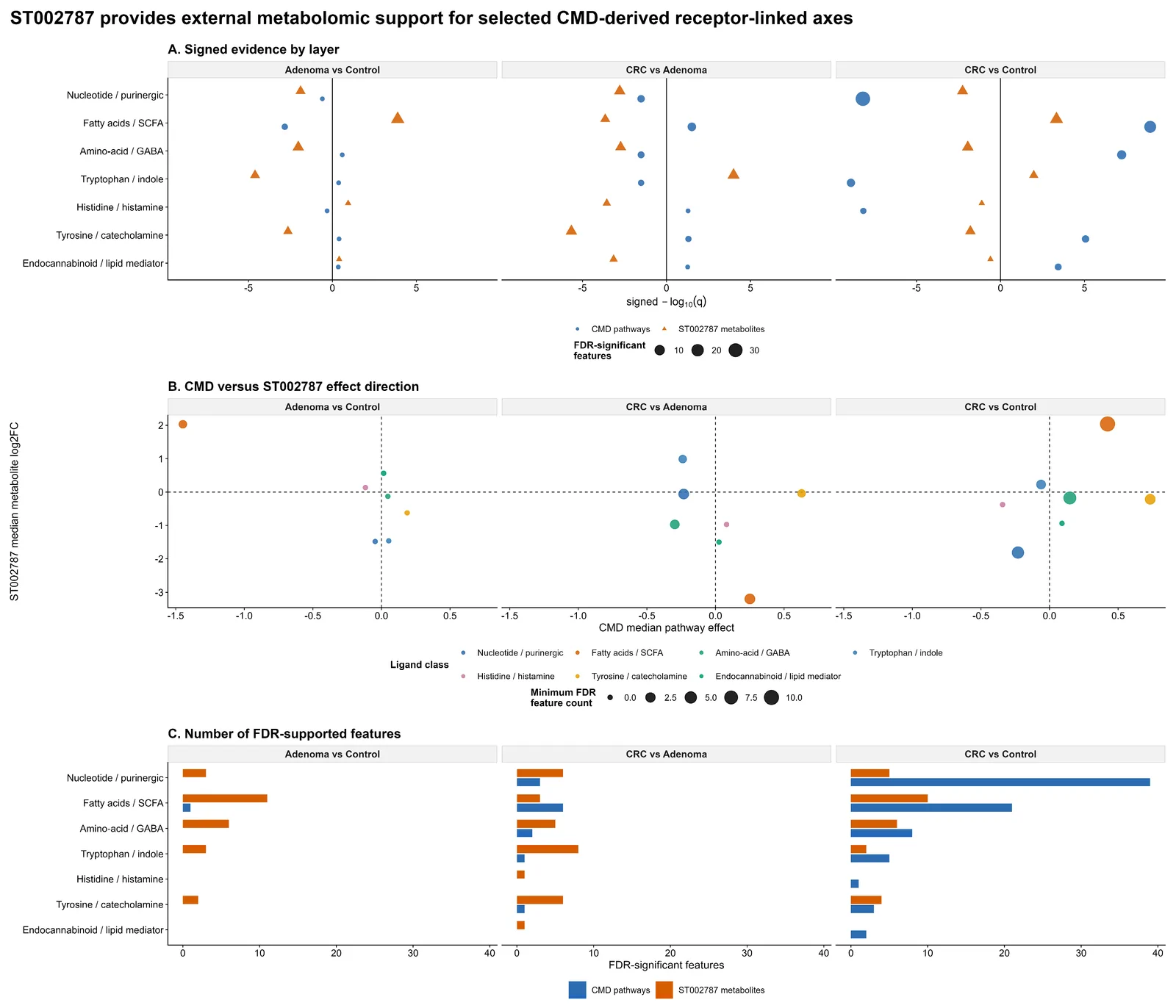
ST002787 external metabolomics support for CMD-derived receptor-linked axes. Panel (**A**) shows signed evidence by layer, with signed −log10(q) plotted separately for CMD pathways and ST002787 metabolites. Panel (**B**) compares CMD median pathway effects with ST002787 median metabolite log2 fold-changes for each biological axis and contrast. Panel (**C**) shows the number of FDR-supported features in each layer.

Other CRC-versus-Control axes showed FDR-supported evidence in one or both layers but less consistent directionality. Amino-acid/GABA had six FDR-supported ST002787 metabolites and eight FDR-supported CMD pathways, but the summarized directions differed. Tryptophan/indole had two FDR-supported ST002787 metabolites and five FDR-supported CMD pathways, also with differing summarized directions. Histidine/histamine had one FDR-supported CMD pathway and no ST002787 metabolite at q < 0.05, although the measured metabolite layer was directionally aligned at the axis level. Tyrosine/catecholamine and endocannabinoid/lipid-mediator axes showed partial or mixed support (Figure 6).

For CRC versus Adenoma, nucleotide/purinergic metabolism and amino-acid/GABA showed directionally concordant FDR-supported evidence across both layers. Nucleotide/purinergic metabolism had six FDR-supported ST002787 metabolites and three FDR-supported CMD pathways, both summarized as lower in CRC versus adenoma. Amino-acid/GABA had five FDR-supported metabolites and two FDR-supported pathways, also aligned in the lower direction. Fatty-acid/SCFA showed FDR-supported evidence in both layers but with discordant summarized direction, with three lower ST002787 metabolites and six CMD pathways summarized predominantly higher in CRC versus adenoma.

The ordered Control-to-Adenoma-to-CRC trend also supported selected receptor-linked axes. Fatty-acid/SCFA signaling showed seven FDR-supported metabolites and 21 FDR-supported metagenomic pathways in the same direction. Nucleotide/purinergic metabolism showed seven FDR-supported metabolites and 39 FDR-supported metagenomic pathways in the same direction. Histidine/histamine signaling showed two FDR-supported metabolites and one FDR-supported metagenomic pathway in the same direction. Amino-acid/GABA-related metabolism showed five FDR-supported metabolites and eight FDR-supported metagenomic pathways in the same summarized direction.

**Figure 7:**
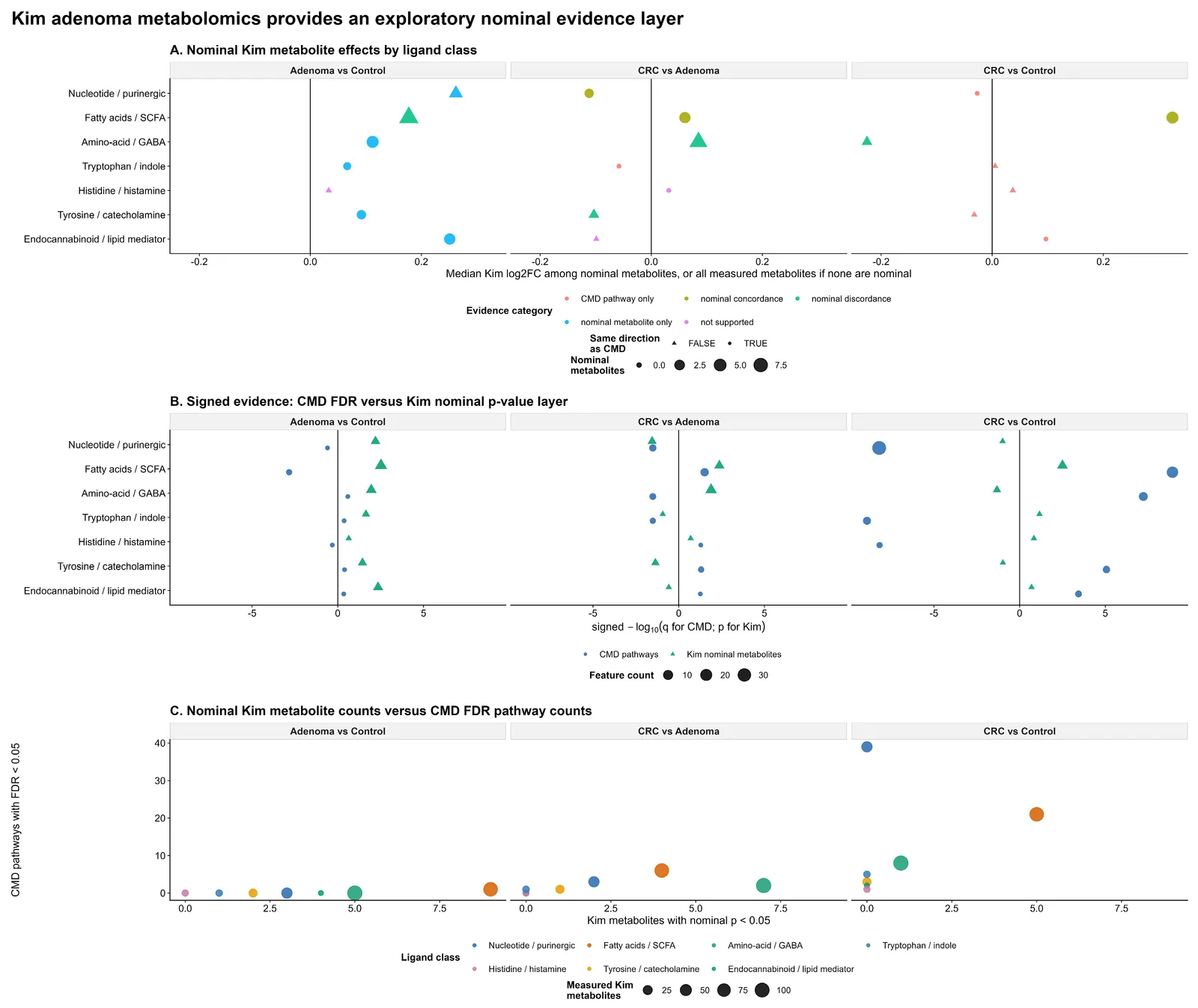
Kim adenoma metabolomics as an exploratory nominal evidence layer. Panel (**A**) shows median Kim metabolite effects by ligand class using nominal *p* < 0.05 metabolites when present, or all measured metabolites when no nominal metabolite was detected. Panel (**B**) compares signed CMD FDR evidence with the Kim nominal *p*-value layer. Panel (**C**) compares nominal Kim metabolite counts with CMD FDR-supported pathway counts.

The Kim adenoma metabolomics dataset provided an exploratory nominal layer. It contained 462 curated metabolite features and 240 samples, including 102 controls, 102 adenomas and 36 CRC samples. No individual metabolite reached BH q < 0.05 in the present reanalysis. At nominal *p* < 0.05, Adenoma versus Control showed nine fatty-acid/SCFA metabolites, all higher in adenoma; four endocannabinoid/lipid-mediator metabolites, all higher in adenoma; five amino-acid/GABA metabolites; three nucleotide/purinergic metabolites; two tyrosine/catecholamine metabolites; and one tryptophan/indole metabolite. These signals were treated as biological context for early neoplasia rather than as confirmatory validation (Figure 7).

In the Kim CRC-versus-Control nominal layer, fatty-acid/SCFA signaling showed five nominal metabolites and was directionally concordant with the CMD pathway effect. Amino-acid/GABA showed one nominal metabolite but was directionally discordant with the CMD axis. The remaining CRC-versus-Control Kim axes were dominated by CMD pathway evidence without nominal metabolite support. Together, ST002787 and Kim provided complementary external metabolomic context: ST002787 gave the FDR-supported external layer, whereas Kim informed adenoma-centered nominal patterns. Neither dataset demonstrates receptor activation, receptor occupancy or individual-level microbiome–metabolite coupling.

To preserve the full categorical evidence summaries, we also provide the matrix-style metabolomics summaries as Supplementary Figures S1 and S2, which classify axis-level agreement between measured metabolite signals and CMD-derived pathway evidence in a compact visual format.

### 2.7. Leave-One-Study-Out Robustness of Selected Findings

Leave-one-study-out refitting supported directional robustness for the selected primary findings. For the top 30 CRC-associated species, 30 of 30 retained the primary effect direction in at least 80% of leave-one-study-out models. The median direction consistency was 1.00, and no feature showed a sign flip. The same pattern was observed for the top 30 unstratified pathways: 30 of 30 met the 80% direction-consistency threshold, the median direction consistency was 1.00, and no sign flip was recorded.

The MMR-mapped pathway sensitivity analysis also supported direction stability among selected pathway findings. Among the top 50 MMR-mapped pathways, 50 of 50 retained the primary effect direction in at least 80% of leave-one-study-out refits. The median direction consistency was 1.00, with no sign flip recorded. These analyses support the direction of selected findings across source-study exclusions, although they do not replace prospective validation in independent cohorts. Because sequencing platform was nested within source study, these leave-one-study-out refits also provide a sensitivity check against findings being dependent on any single study-specific technical configuration, although they cannot isolate sequencing-platform effects from other study-specific characteristics.

## 3. Discussion

### 3.1. Microbial Metabolite–Receptor Axes as Pharmacological Hypotheses

CRC-associated microbiome studies have established reproducible taxonomic and functional signatures but translating these signals into experimentally tractable host biology remains difficult. Our analysis addresses this gap by organizing disease-associated microbial pathways into ligand classes linked to host metabolite-sensing receptor systems. The value of this framework is not that pathway abundance demonstrates receptor activation; it does not. Rather, it converts distributed microbiome associations into defined ligand–receptor modules that can subsequently be tested using targeted metabolomics, receptor assays, pharmacological agonism or antagonism, genetic perturbation and cell-type-resolved host profiling. In the present analysis, fatty-acid/SCFA-lactate and nucleotide/purinergic biology emerged as the most extensively supported receptor-linked modules because they combined substantial metagenomic pathway evidence with independent fecal metabolomic convergence. These modules should not be interpreted as unitary directional signals: their value is to identify receptor-linked biochemical spaces in which individual pathways, metabolites and receptors can be prioritized for subsequent mechanistic testing.

A second point of novelty is the explicit separation of adenoma-concordant, CRC-dominant, and CRC-specific components within the CRC-associated microbiome. This distinction matters because microbial signals in colorectal neoplasia are often discussed as if they were one phenomenon. Our data argue for mixed architecture. Some CRC-associated species and pathways are already directionally shifted in adenoma, even when adenoma-only FDR-significant findings are sparse. Other features are mainly carcinoma-stage signals. This stage-aware structure is relevant because features observed in adenoma as well as CRC may be more informative for early detection or risk stratification, whereas carcinoma-stage features may better reflect tumor-associated ecology or disease burden.

The third innovation is the use of independent fecal metabolomics as a biological convergence analysis for the receptor-axis interpretation. The metabolomics datasets were not paired with the metagenomic samples, so they cannot demonstrate sample-level microbiome–metabolite coupling. Their value is more circumscribed. They test whether the broad ligand axes inferred from metagenomic pathways are also visible as measured fecal metabolite classes in independent colorectal neoplasia cohorts. This external layer was strongest for fatty-acid/SCFA signaling and nucleotide/purinergic metabolism. It also provided adenoma-centered context for lipid mediator, amino-acid, and early metabolic remodeling. Taken together, the data support a cautious model: CRC-associated microbial shifts are not merely functional annotations. A subset maps to metabolite classes for which host cells express dedicated sensing machinery.

### 3.2. Relation to the Established CRC Microbiome Literature

The taxonomic component of the study is consistent with the reproducible CRC stool microbiome signature described by earlier shotgun metagenomic analyses. Cross-cohort studies by Zeller et al., Thomas et al., Wirbel et al., and Yachida et al. established that CRC stool metagenomes often show enrichment of oral-associated anaerobes and pathobionts, together with depletion of common commensal taxa from Firmicutes lineages that contribute to carbohydrate fermentation and mucosal homeostasis. The present species results fit that pattern. CRC samples showed higher *Parvimonas micra*, *Gemella morbillorum*, *Peptostreptococcus stomatis*, *Fusobacterium nucleatum*, *Bacteroides fragilis*, and related taxa, while *Roseburia intestinalis*, *Roseburia faecis*, *Lachnospira eligens*, *Faecalibacterium prausnitzii*, *Ruminococcus bicirculans*, and other commensal lineages were lower. This concordance is important because it shows that the study-adjusted cMD analysis recovers established CRC biology before adding the MMR interpretation layer [1,2,3,4]. The observed overlap with previous studies is primarily confirmatory at the taxonomic level, whereas the contribution of the present analysis lies at a different interpretive level. Earlier CRC microbiome studies established reproducible disease-associated taxa, microbial functions and, in selected cases, organism-specific mechanisms. Here, we instead ask whether community-level functional pathway changes can be organized according to microbial ligand classes linked to host metabolite-sensing receptor systems. The MMR framework therefore builds on the established CRC microbiome signature rather than replacing it, translating a subset of differential microbial functions into defined pathway–ligand–receptor hypotheses for subsequent mechanistic testing. A structured summary of the principal previous studies discussed here, including study populations, core microbial or metabolic findings and reported mechanisms or biological interpretations, is provided in Supplementary Table S1.

The enriched oral and inflammatory anaerobe signal is also mechanistically plausible. *Fusobacterium nucleatum* has been linked experimentally to epithelial adhesion, invasion, beta-catenin signaling, and tumor growth through FadA-mediated interaction with E-cadherin. Additional work showed that its Fap2 protein can engage TIGIT on lymphocytes, providing a route by which a CRC-associated microbe may influence local immune escape [5,6]. Enterotoxigenic *Bacteroides fragilis* provides another example of microbe-driven colorectal tumor biology. *Bacteroides fragilis* toxin triggers a multi-step inflammatory cascade involving IL-17R, NF-kappaB, and STAT3 signaling in colonic epithelial cells in experimental tumorigenesis models [7]. Genotoxic pks-positive *Escherichia coli* further illustrates that microbial functions can leave a durable imprint on the cancer genome. In human intestinal organoids, colibactin exposure produced a mutational pattern that was also detectable in human CRC genomes [8]. These studies do not prove that the taxa identified here caused cancers in the analyzed cohorts. They do show that CRC-associated microbial signatures include organisms and functional programs with credible routes into epithelial signaling, immune modulation, DNA damage, and tumor ecology.

The depleted commensal signal has a different biological meaning. *Faecalibacterium*, *Roseburia*, *Lachnospira*, and related taxa are often associated with fiber fermentation, butyrate production, and anti-inflammatory mucosal states. Their reduction in CRC stool profiles is unlikely to be explained by one pathway alone. It may reflect diet, altered transit time, local inflammation, mucin and nutrient remodeling, luminal oxygenation, antimicrobial exposures, or ecological displacement by tumor-associated organisms. Because harmonized stool-consistency and intestinal-transit data were not available for adjustment, the present analysis cannot determine the extent to which these associations may also reflect disease-associated differences in bowel habit or luminal transit. Still, the consistent depletion of these lineages fits a shift away from a fiber-fermenting, barrier-supportive community. This is relevant to our receptor-centered model because loss of commensal fermentation and gain of alternative fatty-acid, lactate, amino-acid, and nucleotide pathways can change the mixture of ligands available to host metabolite sensors.

### 3.3. Global Structure and the Meaning of Modest Group Separation

The global diversity results should be interpreted carefully. Observed richness and Shannon diversity differed by disease group after study adjustment, whereas Simpson diversity did not. Species and pathway CLR PCA showed extensive overlap between controls, adenoma, and CRC, and study identity explained more variance than disease group in PERMANOVA. However, this does not weaken the feature-level findings. It indicates that colorectal neoplasia does not impose a single large, global restructuring of all stool microbiomes that separates every sample cleanly in low-dimensional ordination space. Instead, the disease signal is distributed across selected taxa and functional pathways and is superimposed on strong between-study variation.

This pattern is expected in public multi-cohort stool metagenomics. Cohorts differ in geography, diet, sample collection, sequencing depth, library preparation, clinical definitions, bowel preparation practices, and metadata completeness. A biological effect that is real but modest can therefore be hard to see in PCA while still being reproducible in study-adjusted feature models. The leave-one-study-out sensitivity analysis strengthens this point. The top CRC-associated species, top unstratified pathways, and selected MMR-mapped pathways retained their direction across study exclusions. Directional robustness does not solve confounding, but it argues against the simplest explanation that the main signals were driven by one large contributing cohort.

The modest global separation also has a biological implication. It suggests that the CRC stool microbiome signature is not a replacement for mechanistic interpretation. A biomarker model might focus on the best discriminating features. Our question was different: among disease-associated features, which biochemical axes might be sensed by host receptors? In that context, overlap in global ordination is not a problem. It reinforces the need for pathway-level and ligand-class analyses rather than relying only on community-wide summary metrics.

### 3.4. Functional Pathway Remodeling and Microbial Metabolite Receptor Biology

The functional results show that the CRC-associated microbiome is organized around multiple metabolic axes. Among unstratified MetaCyc pathways, CRC-versus-control modeling identified a broad pathway signal, with more pathways lower than higher in CRC. Within the strict MMR mapping layer, purine/pyrimidine/nucleotide pathways formed the largest class and were predominantly lower in CRC, whereas SCFA/fatty-acid pathways were fewer but more often higher in CRC. Tryptophan/indole, GABA/amino-acid, and histidine/histamine axes were represented by smaller pathway sets, so their interpretation should remain more cautious. The important point is not that every ligand class shows the same behavior. They do not. The value of the mapping is that it exposes the direction, asymmetry, and biological specificity of the altered microbial ligand space.

Our previous characterization of microbial metabolite receptor gene expression across human malignancies showed altered expression of several metabolite-sensing receptor genes in bulk colorectal tumor tissue [13]. In that pan-cancer analysis, the short-chain fatty acid and lactate receptor genes HCAR1 and HCAR3 were among the most strongly upregulated transcripts in both colon and rectal adenocarcinoma. HCAR1 expression increased 11.0-fold in colon tumors and 15.8-fold in rectal tumors, while HCAR3 expression increased approximately seven-fold in both subsites. The present multi-cohort metagenomic and metabolomic findings add evidence for altered microbial functional potential and independently measured fecal metabolite patterns within a related host–microbiome biological framework. Across the colorectal neoplasia sequence, the gut microbiome shows altered functional potential involving lipid, lactate, and short-chain fatty acid-related pathways, including fermentation routes such as L-lysine fermentation to acetate and butanoate. Independent multi-omics studies support the broader premise that microbiome and metabolome shifts are detectable from early colorectal neoplasia onward. Yachida et al. profiled 616 colonoscopy participants and reported stage-specific fecal metagenomic and metabolomic changes across adenoma, intramucosal carcinoma, and more advanced CRC stages [3]. Clos-Garcia et al. integrated fecal metagenomics and metabolomics and identified CRC-associated lipid changes, including cholesteryl esters and sphingolipids, together with enrichment of CRC-associated taxa and depletion of *Lachnospiraceae* [25]. These studies support coordinated microbial and metabolic remodeling in colorectal neoplasia, although individual SCFA findings vary by diet, tumor stage, sampling matrix, and analytical platform.

The fatty-acid and lactate receptor axis is biologically complex because SCFAs are not uniformly tumor-suppressive or tumor-promoting. Butyrate is the best example. In normal colonocytes it is a major oxidative substrate, whereas in glycolytic cancer cells it can accumulate and act as a histone deacetylase inhibitor. Donohoe et al. showed that the Warburg effect changes the cellular response to butyrate, providing a mechanistic explanation for why the same microbial metabolite may support normal epithelial energetics yet suppress proliferation in cancer cells under specific metabolic states [14]. SCFA receptor signaling adds another layer. Ffar2/Gpr43 signaling has been implicated in fiber-mediated suppression of intestinal carcinogenesis and in maintaining a microbiota configuration associated with tumor protection in mouse models [16]. Lavoie et al. further reported that loss of FFAR2 in dendritic cells promoted colon tumorigenesis in mice by impairing barrier integrity, increasing tumor bacterial load, and promoting CD8+ T cell exhaustion [15]. These studies support the biological relevance of fatty-acid sensing, but they also warn against a simplistic interpretation in which all SCFA-related signals are beneficial.

Recent functional work on lactate sensing strengthens the biological relevance of this axis. He et al. reported that activation of the lactate receptor HCAR1 in colorectal tumor cells induced chemokines including CCL2 and CCL7, which promoted recruitment of immunosuppressive polymorphonuclear myeloid-derived suppressor cells [17]. These functional data indicate that tumor-side lactate-sensing machinery can shape immunosuppressive cell recruitment rather than merely marking a transcriptional state. In the present study, the metagenomic and metabolomic data should be read more conservatively. Lactate within the colorectal tumor microenvironment can arise substantially from host glycolytic metabolism, and the present stool-based analyses cannot distinguish microbial from host-derived lactate or quantify their relative contributions. Accordingly, the HCAR1 literature is used here to establish the biological relevance of lactate sensing in CRC, not to infer that the HCAR1 ligand pool within tumors is predominantly microbially derived. Our data instead support altered microbial functional potential and fecal metabolite patterns within a broader fatty-acid/lactate-related metabolic axis.

In this context, the higher-in-CRC L-lysine fermentation to acetate and butanoate pathway is not simply a butyrate signal in the conventional commensal sense. It may reflect altered metagenomic representation of anaerobic fermentation capacity in a tumor-associated stool ecology, where the producing taxa, co-metabolites, pH, redox state, host uptake, and tumor metabolism all differ from a healthy fiber-fermenting colon. The fecal metabolomics support for fatty-acid/SCFA signaling should therefore be interpreted as axis-level convergence, not as proof that a single SCFA is increased, protective, or pathogenic. This distinction is important. The coexistence of higher- and lower-in-CRC pathways within this class further argues against treating the fatty-acid/SCFA axis as a single directional pharmacological target. Instead, the framework prioritizes pathway-specific and ligand-specific hypotheses within a shared receptor-relevant metabolic domain. The data support remodeling of microbial functional potential and measured fecal metabolites within a ligand class associated with FFAR, HCAR, GPR84, GPR119 and PPAR-related systems. They do not establish which receptor is activated in which host cell type.

### 3.5. Nucleotide Metabolism and Purinergic Signaling

Nucleotide and purinergic pathways reveal a contrasting functional divergence. The present analysis identifies a broad contraction of microbial nucleotide-related pathways, with 36 of 39 mapped functional features lower in CRC samples. Independent fecal metabolomics showed lower abundance of several metabolites assigned to the nucleotide/purinergic axis, providing axis-level convergence with the predominantly lower microbial nucleotide-related pathway signal. This microbial signal is compatible with receptor-level remodeling in our previous host transcriptomic analysis, where colorectal tumors showed altered purinergic receptor expression within cancer hallmark-associated networks. Independent studies support the plausibility of purinergic remodeling in CRC biology. Dillard et al. used RNA-seq and qRT-PCR across CRC cell lines and normal-like colonic epithelial cells and showed that CRC cells express multiple purinergic signaling components, including canonical ADORA and P2RY receptor variants [26]. In rectal cancer, Teng et al. showed that microbiota-mediated nucleotide biosynthesis, particularly involving *Bacteroides vulgatus*, was associated with resistance to neoadjuvant chemoradiotherapy. Nucleoside supplementation or bacterial gavage also protected cancer cells from 5-fluorouracil or irradiation in experimental models [18]. These observations make microbial nucleotide metabolism a biologically credible CRC axis, while not implying that fecal pathway depletion is equivalent to reduced purinergic signaling in tumor tissue.

The direction of the nucleotide signal deserves careful discussion. Lower abundance of microbial nucleotide-related pathways in stool cannot by itself predict whether extracellular ATP, ADP, AMP or adenosine is increased or decreased at the mucosal or tumor interface. Extracellular purine concentrations are jointly determined by host and microbial nucleotide release, biosynthesis and degradation, cellular uptake, epithelial injury, hypoxia, immune-cell turnover and extracellular enzymatic conversion. The present data therefore identify a change in the microbial contribution to nucleotide-related metabolic potential, not the direction of purinergic signaling within tumor tissue. Tumor purinergic activity can consequently be increased, decreased or spatially redistributed even when stool microbial nucleotide pathways are predominantly lower. The value of the axis is therefore not directional concordance between stool pathways and tumor signaling, but the identification of nucleotide metabolism as a strongly altered microbial functional domain that intersects with a host signaling system already implicated in CRC biology.

Host ectonucleotidases are also an important component of this purinergic network. CD39 (ENTPD1) hydrolyses extracellular ATP and ADP to AMP, whereas CD73 (NT5E) converts AMP to adenosine, thereby influencing the balance between nucleotide- and adenosine-mediated signaling [26]. Dillard et al. examined these ectonucleotidases alongside purinergic receptors in CRC cell models, further illustrating that local extracellular purine availability depends on host enzymatic processing as well as on potential microbial contributions [26]. Accordingly, the microbial nucleotide pathways identified here should be interpreted as one component of a broader host–microbial purinergic network rather than as the primary determinant of local receptor activation.

This axis is also clinically interesting because it links microbiome biology to treatment biology. The Teng et al. rectal cancer study is not the same design as ours, but it is conceptually relevant: microbiota-mediated nucleotide biosynthesis influenced response to chemoradiotherapy through effects on nucleoside availability and tumor stress-response programs [18]. Our samples were not therapy-response cohorts and should not be interpreted as predictive of nCRT outcome. Nonetheless, the convergence on nucleotide/purinergic metabolism suggests that this axis deserves attention in future studies that jointly measure fecal metagenomes, luminal metabolites, tumor receptor expression, ectonucleotidase activity, and treatment response.

### 3.6. Tryptophan, Indole, Serotonin-Related Signaling, and Smaller Ligand Axes

Tryptophan and indole signaling represents another host–microbe metabolic axis, but the evidence is more heterogeneous than for fatty-acid/SCFA or nucleotide/purinergic metabolism. Our previous analysis found overexpression of serotonin and indole-related receptor genes in colorectal tumors, including HTR2C and HTR1D, with HTR2C expression 15.1-fold higher in colon tumors and HTR1D expression 10.8-fold higher. In the present metagenomic analysis, the gut microbiome shows reorganization of tryptophan-related functions across the adenoma-to-carcinoma sequence. This includes reduction in core biosynthetic features alongside an increase in selected alternative tryptophan superpathways. Independent fecal metabolomic studies are broadly consistent with altered tryptophan metabolism in CRC. Sun et al. reported changes in fecal tryptophan metabolism, including reduced microbial indole metabolism and associations between indole-related metabolites and specific bacterial taxa [27]. Du et al. reported altered gut microbiome and fecal metabolome profiles in CRC, including increased fecal tryptophan and associations involving *Fusobacterium*, *Ruminococcus bicirculans*, and impaired conversion of tryptophan toward indole-related products [28]. These findings suggest remodeling of the tryptophan axis, but they do not define a single direction of host receptor signaling.

The tryptophan axis should not be reduced to one pathway or one receptor. Microbial indole derivatives can engage AHR, PXR and other host signaling systems, whereas host tryptophan catabolism through the kynurenine pathway can independently influence immune tolerance and tumor biology. The present stool metagenomic analysis measures microbial functional potential and does not measure host kynurenine metabolism; it therefore cannot establish the relative contribution of microbial indole metabolism and host kynurenine metabolism to the CRC microenvironment. The explanatory value of the mapping is consequently narrower: it identifies CRC-associated changes within the microbial branch of tryptophan metabolism that can generate or transform receptor-active indole-related compounds, thereby defining specific pathway–metabolite–receptor relationships for subsequent testing. In vitro receptor assays show that multiple gut microbial tryptophan catabolites can act as AHR agonists, but with different potency and efficacy [29]. The present findings should therefore be interpreted as hypothesis-generating evidence for remodeling of microbial tryptophan/indole potential, not as evidence for a particular direction of AHR activity or altered host kynurenine flux.

The GABA/amino-acid and histidine/histamine axes are biologically plausible but less statistically and structurally developed in the present mapping. GABAergic, glutamatergic, and histaminergic signals can affect epithelial transport, immune cell tone, barrier function, enteric neurons, and stromal behavior. However, the strict metagenomic evidence layer contained few mapped pathways for these classes. The ordinal Control-to-Adenoma-to-CRC metabolomics support for histidine/histamine and amino-acid/GABA-related metabolism is useful, but it should be treated as hypothesis-generating. The same caution applies to tyrosine/catecholamine and endocannabinoid/lipid-mediator signals observed in the external metabolomics summaries. These axes may be important, especially because lipid mediators and catecholamine-related metabolites can shape inflammation, vascular tone, motility, and immune responses. In this dataset, however, they are lower-priority mechanistic leads than fatty-acid/SCFA and nucleotide/purinergic metabolism.

### 3.7. Adenoma-Concordant and Carcinoma-Stage Components

The adenoma-CRC concordance analysis provides a way to separate early-compatible signals from carcinoma-stage signals. Among CRC-associated features, 25% of species and 14% of unstratified pathways were adenoma-concordant, whereas 54% of species and 68% of pathways were CRC-specific by the operational criteria used here. This is biologically credible. Adenomas are heterogeneous lesions that vary in size, histology, dysplasia, location, and molecular subtype. Stool samples also integrate the whole gut lumen, not the local mucosal niche of one polyp. A weak adenoma-only FDR signal is therefore not surprising. The important observation is that the subset of CRC-associated features with nominal adenoma support did not show discordant directions and was detectable in adenoma as well as CRC. This cross-sectional pattern does not establish whether those features preceded lesion development, were induced by the adenoma, or reflected a pre-existing microbial state.

The early concordant species require cautious interpretation. *Ruminococcus bicirculans*, for example, was lower in adenoma and appears in the representative concordance pattern. This species has been discussed in fecal metabolomics studies in relation to tryptophan and indole metabolism, but its role is not established as protective or causal. More generally, reduced commensal fermenters in adenoma may indicate early changes in diet-linked ecology, mucosal inflammation, or altered luminal chemistry rather than direct adenoma promotion. Conversely, oral-associated anaerobes and inflammatory taxa that emerge mainly in CRC may reflect conditions created by the tumor, including bleeding, necrosis, altered oxygen gradients, local immune suppression, and new nutrient niches. The classification is therefore best viewed as a cross-sectional stage-pattern map of association signals, not as a temporal lineage or model of adenoma-to-carcinoma progression.

Recent evidence from an independent prospective setting supports the interpretation that colorectal neoplasia can be associated with a durable microbial-metabolic state rather than a transient alteration restricted to the presence of a visible lesion. Nogal et al. analyzed stool metagenomes and fecal metabolomes from 354 women with prior adenoma removal and 354 matched adenoma-free controls in the Nurses’ Health Study II, with stool collected on average 12 years after adenoma removal [12]. They found persistent differences in gut microbial composition and fecal metabolites, and the post-adenoma microbiome partially resembled CRC-associated microbial profiles. These findings also provide an alternative interpretation of the adenoma-concordant signals identified here: some may represent persistent or pre-existing host–microbial states associated with neoplasia susceptibility rather than microbial changes initiated by the adenoma itself. That study remained observational and addressed a different biological window: long-term post-polypectomy risk rather than active neoplastic progression. It is nevertheless consistent with our observation that CRC-associated metagenomic features are partly organized along the control-adenoma-CRC continuum. The receptor-informed layer proposed here adds a different question: whether altered microbial functional potential can be organized into candidate ligand classes for host epithelial, immune, and stromal sensing pathways.

The adenoma findings also fit earlier multi-omics work showing that metabolic changes can appear before invasive cancer. Kim et al. profiled fecal metabolites in colorectal adenoma and CRC and reported adenoma-associated metabolomic signatures linked to gut microbiota and early CRC pathogenesis [10]. More recently, Sun et al. analyzed plasma and fecal metabolomes across normal, adenoma, and CRC groups in multiple cohorts, identifying progression-associated metabolites and experimental effects of selected metabolites such as oleic acid and allocholic acid in CRC models [11]. These studies strengthen the idea that the adenoma-to-CRC transition includes metabolic remodeling, but they also show that the relevant metabolites may be lipids, bile acids, amino-acid derivatives, and other compound classes rather than one microbial product family. Our MMR framework is compatible with that complexity because it organizes microbial pathways by ligand classes and receptor systems instead of forcing a single-metabolite explanation.

### 3.8. External Fecal Metabolomics and Biological Interpretation

The external metabolomics results should be read as biological triangulation. ST002787 provided the clearest FDR-supported evidence for fatty-acid/SCFA and nucleotide/purinergic axes, with measured metabolites and metagenomic pathways supporting the same broad directions in CRC-versus-control and ordered Control-to-Adenoma-to-CRC analyses. The Kim dataset did not yield individual FDR-significant metabolites under our reanalysis, but nominal axis-level signals were present in adenoma, especially for fatty-acid/SCFA, endocannabinoid/lipid-mediator, and amino-acid/GABA-related metabolism. This difference between datasets is expected. Metabolomics studies differ in sample handling, extraction chemistry, chromatographic platform, feature annotation, statistical power, and disease composition. A lack of FDR significance in one reanalysis does not erase the biological pattern, but it lowers the strength of inference.

Several independent fecal metabolome studies support the broader premise that CRC is accompanied by coordinated microbial and metabolic remodeling. Sinha et al. reported strong microbe–metabolite correlations in CRC feces, involving *Enterobacteriaceae* and *Actinobacteria*, and found that metabolites mediated some CRC associations with taxa such as *Fusobacterium* and *Porphyromonas* [9]. Clos-Garcia et al. combined fecal metagenomics and metabolomics and described CRC-associated microbial changes together with alterations in lipids and related metabolites. Yachida et al. showed that stage-specific metagenomic and metabolomic phenotypes can be detected across the colorectal neoplasia sequence [3]. These studies support the rationale for integrating pathway-inferred ligand classes with measured metabolite axes, although they do not remove the need for paired microbiome–metabolome–receptor datasets.

A key interpretive boundary is that fecal metabolite abundance is not the same as receptor exposure at the tumor surface. Feces represent the distal luminal output of digestion, microbial metabolism, host secretion, absorption, transit time, and sample handling. Tumor cells, immune cells, fibroblasts, endothelial cells, and neurons experience local gradients within mucus, tissue, and interstitial compartments that stool measurements only approximate. Some metabolites may be rapidly absorbed or transformed before appearing in feces. Others may accumulate in stool without reaching the tumor in active concentrations. For this reason, the metabolomics layer strengthens biological plausibility at the axis level but does not demonstrate receptor occupancy or downstream signaling.

### 3.9. Implications for Host–Microbiome Biology in CRC

Viewed alongside prior host receptor analyses, the present findings support a broader model of host–microbiome metabolic coordination in colorectal neoplasia. The data do not show receptor activation, receptor protein abundance, or cell-type-specific signaling. They do show that reproducible microbial pathway shifts map to ligand classes connected to host metabolite-sensing receptor families. The strongest evidence in this study involves fatty-acid/SCFA and nucleotide/purinergic biology, with more tentative signals in tryptophan/indole, amino-acid/GABA, histidine/histamine, lipid mediator, and catecholamine-related metabolism. This supports a model in which microbial functional remodeling and previously observed host receptor remodeling converge on related metabolic-sensing axes, rather than a model in which every altered pathway directly activates a corresponding receptor. Accordingly, the pathway component of the framework should be interpreted as altered biochemical capacity rather than evidence that the corresponding metabolites were actively produced or delivered to host receptors in the analyzed individuals.

This model changes how microbiome findings can be prioritized experimentally. Rather than testing every altered species or pathway individually, future work can focus on matched ligand–receptor modules. Fatty-acid/SCFA and lactate pathways can be tested together with HCAR1, HCAR3, FFAR2, FFAR3, GPR84, GPR119, and PPAR-related activity in epithelial and immune compartments. Nucleotide/purinergic pathway shifts can be evaluated with ADORA, P2RY, P2RX, CD39, CD73, and extracellular ATP/adenosine measurements. Tryptophan/indole pathway changes can be paired with AHR reporter activity, indole derivative quantification, and serotonin receptor expression. Such experiments would move the field from association to mechanism by asking which microbial ligand classes are present, which host cells can sense them, and which signaling outcomes follow.

From a pharmacological perspective, organizing microbiome-associated changes into ligand–receptor modules provides a more tractable starting point than considering individual differential taxa in isolation. Candidate intervention strategies could act at several levels, including microbial production or degradation of metabolites, dietary or microbial manipulation of substrate availability, targeted metabolite supplementation or sequestration, and modulation of host metabolite-sensing receptors or their downstream signaling pathways. The fatty-acid/SCFA-lactate and nucleotide/purinergic axes identified here are particularly suitable for such investigation because both microbial metabolic potential and independently measured fecal metabolites support their alteration in colorectal neoplasia. These findings do not establish these axes as therapeutic targets. Rather, they prioritize defined host–microbiome interfaces for pharmacological testing of directionality, receptor engagement, efficacy, selectivity and safety.

### 3.10. Limitations

The main limitation is causal inference. This was a retrospective analysis of public stool metagenomes and independent fecal metabolomics datasets. It cannot determine whether microbial changes preceded neoplasia, resulted from tumor-associated ecological changes, or reflected shared upstream exposures such as diet, medication, inflammation, bowel habits, or screening context. The adenoma-concordant features are compatible with early involvement, but they do not prove temporal precedence. The CRC-specific and CRC-dominant features may include both drivers and passengers. The correct interpretation is that the data identify reproducible association patterns and biologically coherent ligand axes that require prospective and experimental validation.

A second limitation is the nature of metagenomic functional potential. HUMAnN/MetaCyc pathway abundance reflects inferred gene/pathway capacity from shotgun metagenomic profiles. It is not microbial transcription, enzyme activity, metabolic flux, or metabolite concentration. Pathways can be present but inactive, and activity can be regulated by substrate availability, microbial strain variation, redox state, pH, cross-feeding, and host absorption. The MMR mapping layer is therefore an interpretation of potential ligand-generating or ligand-transforming functions, not a direct measurement of receptor ligands. This boundary is especially important for SCFA, tryptophan, and nucleotide pathways, where pathway names can imply biochemical capacity but not net luminal production.

A third limitation is cohort heterogeneity and incomplete covariate control. Study adjustment reduced source-study confounding, and leave-one-study-out analysis supported directional robustness for selected findings. However, the contributing public datasets differed in the availability, completeness and coding of demographic, clinical and lifestyle variables, including diet, medication exposures, antibiotic use, proton-pump inhibitor and metformin use, bowel preparation, stool consistency, intestinal transit, tumor location, histology, stage, molecular subtype and inflammatory markers. Consequently, a uniform multivariable adjustment strategy incorporating these factors could not be applied across all cohorts. Residual individual-level confounding therefore remains possible, and the observed associations cannot be attributed uniquely to colorectal neoplasia.

A fourth limitation is the independence of the metagenomic and metabolomic datasets. Independent metabolomics provided axis-level biological convergence for selected MMR axes, but the samples were not paired with the CMD metagenomes. We therefore could not estimate sample-level correlations between microbial pathways and metabolites, nor could we test whether a specific species contributed to a specific metabolite in the same individual. The metabolomics analyses were intentionally summarized at the biological-axis level. This is useful for external convergence, but it is weaker than integrated multi-omics from the same stool samples and still weaker than spatially resolved measurements from tumor-adjacent mucosa.

A fifth limitation is receptor inference. The receptor map was curated from ligand-class biology and prior host transcriptomic work. It does not show receptor protein abundance, receptor localization, ligand binding, intracellular signaling, or cell-type specificity in the current samples. Tumor tissue contains epithelial cells, fibroblasts, endothelial cells, neurons, myeloid cells, lymphocytes, and other stromal components. Bulk transcriptomic receptor signals can reflect changes in cell composition as well as regulation within a cell type. A strong future test of the model would combine stool shotgun metagenomics, targeted fecal and mucosal metabolomics, single-cell or spatial transcriptomics of MMR-expressing host cells, and functional assays of receptor activation.

Finally, the MMR ligand-class framework is necessarily selective and should not be interpreted as a comprehensive map of CRC-associated microbial metabolism. It prioritizes the predefined receptor-linked ligand classes examined here, whereas CRC microbiome biology also includes microbial adhesion, toxins, genotoxins, bile-acid metabolism, choline/TMA-related metabolism, sulfur metabolism, immune pattern recognition, bacteriophages, fungi and strain-level variation. Some of these mechanisms are only partly captured by unstratified MetaCyc pathways, and others are not captured well at all. In addition, the use of unstratified pathway abundance means that the present analysis cannot determine whether a pathway-level association was driven predominantly by a single taxon or reflected distributed contributions across the microbial community. Taxon-stratified functional analysis would be required to resolve these organism-specific contributions. The same limitation applies within species: species-level taxonomic associations cannot distinguish functionally divergent strains, such as pks-positive from pks-negative *Escherichia coli* or enterotoxigenic from non-toxigenic *Bacteroides fragilis*. Consequently, strain-specific mechanisms should not be inferred from the present species or unstratified pathway associations [7,8]. The framework should therefore be viewed as one biologically motivated lens, not as a complete model of microbiome involvement in CRC.

## 4. Materials and Methods

### 4.1. Study Design, Data Source and Cohort Definition

We performed a retrospective multi-study analysis of publicly available stool shotgun metagenomes from curatedMetagenomicData (CMD) version 3.14.0, accessed in R version 4.4.0 through Bioconductor and ExperimentHub. curatedMetagenomicData provides manually curated metadata and standardized taxonomic and functional profiles for human metagenomic studies. Candidate samples were restricted to stool specimens annotated as control, adenoma or colorectal cancer (CRC). Studies were eligible when they included at least 10 control samples and at least 10 CRC samples. Adenoma samples were retained when present within eligible studies. This secondary analysis used de-identified public data and generated no new human participant data.

The final dataset contained 1534 stool metagenomes from the following 11 curatedMetagenomicData study identifiers: YachidaS_2019 [3], ZellerG_2014 [4], WirbelJ_2018 [2], YuJ_2015 [22], VogtmannE_2016 [23], FengQ_2015 [20], HanniganGD_2017 [21], ThomasAM_2019_c, ThomasAM_2018a, ThomasAM_2018b [1] and GuptaA_2019 [24]. These curated identifiers correspond to published colorectal cancer or colorectal neoplasia metagenomic resources, including original cohort studies and cross-cohort CRC metagenomic analyses. Disease groups were encoded as Control, Adenoma and CRC. For ordinal analyses, these groups were coded as 0, 1 and 2, respectively. Study identifier was retained as the main blocking variable and included in adjusted models.

### 4.2. Species and Pathway Feature Layers

Two curatedMetagenomicData feature layers were analyzed. The taxonomic layer was the relative_abundance matrix, corresponding to MetaPhlAn3 species-level taxonomic profiles. The functional layer was the pathway_abundance matrix, corresponding to HUMAnN3 MetaCyc pathway abundance profiles generated within the bioBakery 3 framework. The main functional analyses used unstratified MetaCyc pathways, which summarize community-level pathway abundance without resolving the signal to individual contributing taxa. This choice was deliberate because the primary analytical question was whether disease-associated microbial functional potential, irrespective of the taxonomic source contributing that potential, mapped to metabolite ligand classes relevant to host receptor systems. Taxon-stratified pathway profiles address a related but different question concerning which organisms contribute individual pathway signals and were therefore outside the scope of the primary receptor-axis analysis. Accordingly, the unstratified pathway profiles should be interpreted as community-level functional associations. They do not identify the species or strain contributing a given pathway signal and cannot resolve strain-specific gene-content differences or virulence determinants.

Throughout this manuscript, metagenomic functional potential refers to pathway abundance inferred from shotgun metagenomic profiles. It does not denote measured metabolite concentration, pathway flux, microbial transcriptional activity, host receptor expression, receptor occupancy or receptor activation.

### 4.3. Preprocessing, Prevalence Filtering and CLR Transformation

Feature matrices were aligned to the final metadata by sample identifier before filtering. Species and pathway features were retained when they had non-zero abundance in at least 10% of samples in the final analysis set. This threshold retained 217 species-level taxonomic features from 927 raw relative-abundance features and 7725 pathway-abundance features from 38,690 raw pathway features. The main unstratified MetaCyc pathway analysis retained 420 pathways.

For each feature layer, a pseudocount equal to half of the minimum positive value in that layer was added before centered log-ratio (CLR) transformation. The exported preprocessing summary recorded pseudocounts of 2.5 × 10^−5^ for the species relative-abundance layer and 3.619230 × 10^−9^ for the full pathway-abundance layer. After pseudocount addition, each sample vector was transformed by subtracting the sample-wise log geometric mean from the log abundance of each retained feature. CLR transformation was used because taxonomic and pathway abundance profiles are compositional.

### 4.4. Alpha-Diversity and Global Structure Analyses

Species-level alpha diversity was calculated from the filtered species relative-abundance matrix before CLR transformation. Observed species richness was defined as the number of retained species with non-zero abundance in each sample. Shannon and Simpson diversity were computed with vegan version 2.6-10. Disease-group differences in alpha diversity were tested using linear models that included disease_group and study_name, and the disease_group term was evaluated in study-adjusted ANOVA models.

Global species and pathway structure were assessed by principal component analysis of the corresponding CLR-transformed matrices. Euclidean distances were calculated from CLR profiles for multivariable testing. PERMANOVA was performed using vegan::adonis2 with 999 permutations. Unadjusted models included disease_group alone. Study-aware models included study_name followed by disease_group, and these models were used for the main interpretation because source cohort effects were expected in public multi-study metagenomic data.

### 4.5. Study-Adjusted Differential Species and Pathway Modeling

Differential feature analyses were performed on CLR-transformed feature matrices using limma version 3.62.2. Pairwise disease-group contrasts used a no-intercept group design with study_name included as a covariate. The primary contrasts were CRC versus Control, Adenoma versus Control and CRC versus Adenoma. A separate ordinal model tested the Control-to-Adenoma-to-CRC gradient using disease_ordinal and study_name.

Empirical Bayes moderation used eBayes with trend = TRUE and robust = TRUE. Model outputs included CLR effect estimates, moderated t statistics, nominal *p*-values, Benjamini–Hochberg FDR-adjusted q-values and approximate 95% confidence intervals. Multiple testing was controlled within each feature layer and contrast using the Benjamini–Hochberg procedure [30]. Features with q < 0.05 were considered FDR-significant. The primary metagenomic models used study identifier as the common adjustment variable across cohorts. Additional demographic, clinical and lifestyle covariates were not included in the prespecified primary models because their availability, completeness and coding differed across the contributing source datasets, preventing a uniform multivariable adjustment strategy across all cohorts. Sequencing platform was additionally examined as a potential technical covariate during sensitivity assessment. In the final harmonized dataset, sequencing platform was nested within source study and was therefore not independently estimable in models already containing study identifier. We therefore did not interpret study adjustment as demonstrating the absence of platform effects. Stool-consistency or Bristol Stool Scale information did not provide usable observations in the harmonized dataset, and no consistently available harmonized measure of intestinal transit was available across the included studies. These variables therefore could not be incorporated into the study-adjusted models.

### 4.6. MMR Ligand-Class and Receptor-Axis Curation

The microbial metabolite receptor (MMR) interpretation layer was built to connect CRC-associated microbial pathways with ligand classes relevant to host metabolite-sensing receptor systems. The receptor-family framework was based on a curated MMR map and prior pan-cancer work on microbial metabolite receptor expression [13]. The mapped host receptor systems included ADORA/P2RY/P2RX purinergic receptors; FFAR, HCAR, GPR84, GPR119 and PPAR-related fatty-acid and SCFA systems; AHR, AHRR, GPR35 and selected serotonin-related systems; GABA receptor systems; HRH histamine receptor systems; ADRA, ADRB and DRD catecholamine-related receptor systems; and endocannabinoid or lipid-mediator receptors. The seven ligand classes were prespecified from this receptor-centered framework and were not intended to provide an exhaustive representation of CRC-associated microbial metabolism. Accordingly, other microbiome-related metabolic processes relevant to CRC, including bile-acid metabolism, choline/TMA-related metabolism and sulfur metabolism, were not incorporated into the primary MMR axis analysis unless they mapped to the predefined receptor-linked ligand classes. Within this framework, the fatty-acid/SCFA category was an umbrella receptor-oriented class rather than a claim of physiological equivalence among its constituent metabolites. Acetate, propionate, butyrate, lactate and other fatty-acid-related metabolites can have distinct biological effects; therefore, the category-level summary was used for organization, while mechanistic interpretation remained dependent on the individual pathway or metabolite.

Unstratified MetaCyc pathway names, identifiers and searchable annotations were curated against prespecified ligand-class rules. Pathway assignments distinguished direct ligand-related evidence from broader ligand-class evidence. Direct ligand-related evidence was assigned when a pathway named, produced, degraded or transformed a relevant ligand or close biochemical precursor. Ligand-class evidence was assigned when a pathway mapped to a broader biochemical class linked to one of the receptor axes. Pathway counts, FDR-supported directions, pathway-level CLR effects, q-values and receptor annotations for each axis were summarized accordingly.

### 4.7. External Fecal Metabolomics Analysis

Independent fecal metabolomics datasets were analyzed to assess whether the metagenomic MMR ligand-axis framework was reflected in measured metabolites. Data available at the NIH Common Fund’s National Metabolomics Data Repository (NMDR) website, the Metabolomics Workbench [31], from the study with ID: ST002787 and Project DOI: 10.21228/M85X48, were used as the primary external dataset. It included 102 fecal samples, comprising 30 controls, 37 colorectal adenoma samples and 35 CRC samples. Positive and negative ion-mode matrices were imported separately, assigned ion-mode labels and combined after metabolite-name harmonization. Disease labels were recoded to Control, Adenoma and CRC.

For ST002787, metabolites detected in at least 50% of samples in at least one disease group were retained. The analysis pipeline specified replacement of missing values with half of the minimum positive value for each metabolite before log2 transformation. However, inspection of the imported ST002787 analyte matrices showed that, after exclusion of blank non-analyte rows, no analyte abundance observations were missing and therefore no values required imputation. As a sensitivity check, the differential analysis was repeated using available-case processing without imputation and under a more stringent metabolite-detection criterion. These analyses were used to evaluate whether the FDR-supported metabolomic findings depended on the prespecified missing-value procedure. For each metabolite and ion mode, limma models tested CRC versus Control, Adenoma versus Control, CRC versus Adenoma and the ordered Control-to-Adenoma-to-CRC trend [32]. Models included disease group and, when available, age and sex. Positive and negative ion-mode results for duplicate metabolite names were collapsed at the result level by retaining the lower FDR result for each contrast.

The Kim et al. adenoma fecal metabolomics dataset, accessed through The Curated Gut Microbiome Metabolome Data Resource, was reanalyzed as an adenoma-centered context dataset [10]. The processed curated data contained 462 metabolite features and 240 samples, comprising 102 controls, 102 adenomas and 36 carcinomas, which were recoded as CRC for consistency with the metagenomic analysis. These values were analyzed on the processed scale provided by the source resource. The same disease contrasts and ordinal trend model were applied. Benjamini–Hochberg FDR values were retained. Because no Kim metabolite reached q < 0.05 in this reanalysis, the Kim layer was summarized as an exploratory nominal *p* < 0.05 screen rather than as confirmatory metabolomic evidence.

Metabolite names and RefMet or curated annotations were mapped to the same reader-facing MMR-relevant axes used for the metagenomic pathway interpretation: fatty-acid/SCFA signaling, nucleotide/purinergic metabolism, amino-acid/GABA-related metabolism, tryptophan/indole signaling, histidine/histamine signaling, tyrosine/catecholamine-related metabolism and endocannabinoid/lipid-mediator signaling. Because the metabolomics and metagenomic cohorts were independent, the analysis tested convergence at the biological-axis level rather than individual-level microbiome–metabolite correlations.

### 4.8. Adenoma-CRC Concordance and Stage-Pattern Classification

The adenoma-CRC concordance analysis was designed as a descriptive classification of CRC-associated features according to whether the same features also showed concordant evidence in adenoma. Species and unstratified pathways were classified by combining Adenoma versus Control, CRC versus Control, CRC versus Adenoma and ordinal Control-to-Adenoma-to-CRC effects. The main classification summary was restricted to features that were FDR-significant in CRC versus Control.

Adenoma-concordant CRC signals required an FDR-significant CRC association, concordant adenoma and CRC effect directions, nominal adenoma support (*p* < 0.05), and an absolute adenoma CLR effect of at least 0.05. CRC-dominant signals required a strict CRC association, a strict CRC versus Adenoma association and a CRC versus Adenoma effect direction matching the CRC versus Control effect. CRC-specific signals required a strict CRC association, no nominal adenoma support and an absolute CRC CLR effect of at least 0.10. The adenoma-concordant category was therefore a descriptive subclassification of FDR-supported CRC features and should not be interpreted as an independently FDR-significant adenoma signature.

### 4.9. Leave-One-Study-Out Robustness Analysis

Leave-one-study-out robustness was assessed by refitting the CRC versus Control model after excluding each study in turn. This analysis was performed for the top 30 CRC-associated species, the top 30 CRC-associated unstratified pathways and the top 50 MMR-mapped pathways. Direction consistency was defined as the fraction of refits that retained the primary effect direction. Features were considered directionally robust when the primary direction was retained in at least 80% of refits.

### 4.10. Software and Figure Generation

Analyses were performed in R version 4.4.0. The main attached packages were curatedMetagenomicData 3.14.0, TreeSummarizedExperiment 2.14.0, SummarizedExperiment 1.36.0, limma 3.62.2, vegan 2.6-10, ggplot2 4.0.2, dplyr 1.1.4, tidyr 1.3.1, readr 2.1.5, stringr 1.5.1, tibble 3.2.1, broom 1.0.7, patchwork 1.3.2, scales 1.4.0, igraph 2.1.4, ggalluvial 0.12.5 and fs 1.6.5. Figures were generated from exported analysis tables using ggplot2 and patchwork.

## 5. Conclusions

Overall, three main conclusions emerge from this study. First, CRC is associated with reproducible species and pathway changes across 11 stool metagenomic cohorts, while a subset of CRC-associated features also shows concordant evidence in adenoma. Second, organizing altered microbial pathways according to receptor-linked ligand classes identifies fatty-acid/SCFA-lactate and nucleotide/purinergic metabolism as the most strongly supported axes, with independent fecal metabolomic convergence. Third, this receptor-centered framework reduces a complex CRC microbiome signal to defined microbial pathway–metabolite–receptor relationships that can be tested prospectively using paired multi-omics, spatial host profiling and receptor-level functional experiments.

## Figures and Tables

**Table 1:** Study-level sample counts in the final analysis set.

Study	Control	Adenoma	CRC	Total
YachidaS_2019	246	67	258	571
YuJ_2015	38	0	74	112
WirbelJ_2018	65	0	60	125
ZellerG_2014	61	42	53	156
VogtmannE_2016	58	0	52	110
FengQ_2015	16	47	46	109
ThomasAM_2019_c	40	0	40	80
ThomasAM_2018b	28	0	32	60
GuptaA_2019	30	0	30	60
ThomasAM_2018a	14	27	29	70
HanniganGD_2017	28	26	27	81
Total	624	209	701	1534

## Data Availability

The data presented in this study are available in curatedMetagenomicData (CMD) version 3.14.0 through Bioconductor and ExperimentHub at https://bioconductor.org/packages/curatedMetagenomicData/ (accessed on 1 October 2026); in the NIH Common Fund National Metabolomics Data Repository (NMDR), Metabolomics Workbench, at https://www.metabolomicsworkbench.org (accessed on 1 October 2026), reference number ST002787, Project DOI 10.21228/M85X48; and in the Curated Gut Microbiome Metabolome Data Resource at https://github.com/borenstein-lab/microbiome-metabolome-curated-data/tree/main/data/processed_data/KIM_ADENOMAS_2020 (accessed on 1 October 2026), reference number KIM_ADENOMAS_2020. These data were derived from the following resources available in the public domain: curatedMetagenomicData (CMD) version 3.14.0, available through Bioconductor and ExperimentHub at https://bioconductor.org/packages/curatedMetagenomicData/ (accessed on 1 October 2026); Metabolomics Workbench, https://www.metabolomicsworkbench.org (accessed on 1 October 2026), Study ID ST002787, Project DOI 10.21228/M85X48; and the Curated Gut Microbiome Metabolome Data Resource, https://github.com/borenstein-lab/microbiome-metabolome-curated-data/tree/main/data/processed_data/KIM_ADENOMAS_2020 (accessed on 1 October 2026).

## Supplementary Materials

The following supporting information can be downloaded at https://www.mdpi.com/article/10.3390/ph19101608/s1. Figure S1: Categorical ST002787 evidence matrix; Figure S2: Categorical Kim adenoma evidence matrix; Figure S3, Source-study structure of species and pathway CLR principal component analyses; Table S1: Selected studies informing the CRC microbiome, metabolite and host-receptor framework.

## Abbreviations

The following abbreviations are used in this manuscript:

ADORA	Adenosine A receptor family
ADRA	Adrenergic α receptor family
ADRB	Adrenergic β receptor family
AHR	Aryl hydrocarbon receptor
AHRR	Aryl hydrocarbon receptor repressor
CLR	Centered log-ratio
CMD	Curated Metagenomic Data
CRC	Colorectal cancer
FFAR	Free fatty acid receptor family
GABA	Gamma-aminobutyric acid
GPR	G protein-coupled receptor
HCAR	Hydroxycarboxylic acid receptor family
HRH	Histamine receptor family
MMR	Microbial metabolite receptor
NMDR	National Metabolomics Data Repository
PCA	Principal component analysis
SCFA	Short-chain fatty acid
