# Supplementary material for: Colorectal Cancer-Associated Microbiome Remodeling Implicates Host Microbial Metabolite–Receptor Axes
Source: Pharmaceuticals (Basel). 2026 Oct 10;19(10):1608. doi: 10.3390/ph19101608 (PMC13708536; doi:10.3390/ph19101608)
Supplement: Supplementary file 1 [file pharmaceuticals-19-01608-s001.zip › Supplememtary figure legends.pdf]

Supplementary Figure S1. Categorical ST002787 evidence matrix. Tile colors summarize agreement between FDR-supported fecal metabolite signals from ST002787 and FDR-supported microbial pathway signals within each MMR-relevant biological axis. The matrix should be interpreted as a compact evidence classification, whereas Figure 6 provides the quantitative signed-evidence display.

Supplementary Figure S2. Categorical Kim adenoma evidence matrix. Tile colors summarize nominal metabolite signals from the Kim adenoma metabolomics dataset and their relationship to CMD pathway axes. This matrix is exploratory because the Kim reanalysis did not identify individual metabolites at BH $q < 0.05$.

Supplementary Figure S3. Source-study structure of species and pathway CLR principal component analyses. The same CLR PCA coordinates displayed in Figure 2 are shown according to source study for (A) species-level profiles and (B) unstratified MetaCyc pathway profiles. Point shape denotes disease group. The visualization demonstrates the substantial between-study structure present in the pooled dataset and is provided for transparency rather than as evidence that study- or platform-associated effects were removed. Sequencing platform was nested within source study in the analyzed dataset and therefore could not be independently estimated in models already containing study identifier.
