# Supplementary material for: Colorectal Cancer-Associated Microbiome Remodeling Implicates Host Microbial Metabolite–Receptor Axes
Source: Pharmaceuticals (Basel). 2026 Oct 10;19(10):1608. doi: 10.3390/ph19101608 (PMC13708536; doi:10.3390/ph19101608)
Supplement: Supplementary file 1 [file pharmaceuticals-19-01608-s001.zip › Supplementary Table S1.pdf]

Supplementary Table S1. Selected studies informing the CRC microbiome, metabolite and host-receptor framework

Reference	Study subjects/experimental model	Core microbial taxa or metabolic axis	Principal finding relevant to the present study	Reported mechanism or biological interpretation
**Thomas et al., 2019 [1]**	Human multi-cohort stool shotgun metagenomics; five public datasets, two new cohorts and two validation cohorts (969 faecal metagenomes in total).	Cross-cohort CRC taxonomic and functional signatures; choline degradation.	Identified reproducible CRC-associated microbial signatures across cohorts and functional differences that included choline degradation.	Supports reproducible taxonomic and functional remodelling in CRC. This is association-level evidence rather than a direct demonstration of host receptor activation.
**Wirbel et al., 2019 [2]**	Human meta-analysis of eight geographically and technically diverse stool shotgun metagenomic studies (CRC meta-analysis n = 768), with independent validation.	Core CRC-associated microbial species; protein/mucin catabolism, carbohydrate degradation and inferred secondary bile-acid production.	Identified a globally reproducible CRC metagenomic signature, with enriched protein and mucin catabolism genes and depleted carbohydrate degradation genes.	Establishes cross-cohort reproducibility of CRC taxonomic and functional microbiome changes and shows that altered microbial metabolism extends beyond individual taxa.
**Yachida et al., 2019 [3]**	Human colonoscopy cohort of 616 participants spanning controls, multiple polypoid adenomas, intramucosal carcinoma and more advanced CRC.	Stage-associated microbiome and metabolome changes, including *Fusobacterium nucleatum*, early-stage taxa, amino acids and bile acids.	Reported distinct faecal metagenomic and metabolomic patterns across colorectal neoplasia stages, including alterations detectable in early lesions.	Supports coordinated microbial and metabolic remodelling across colorectal neoplasia but does not itself establish receptor activation or temporal causality.
**Zeller et al., 2014 [4]**	Human CRC and tumour-free controls; discovery cohort of 156 participants with validation in independent populations (n = 335).	CRC-associated stool microbial markers; inferred shift from fibre degradation toward host carbohydrate/amino-acid utilisation and increased lipopolysaccharide metabolism.	Identified taxonomic markers that distinguished CRC from controls and retained performance in independent cohorts, including early-stage disease.	Provides an established CRC taxonomic and functional background against which the present receptor-axis framework can be interpreted.
**Gur et al., 2015 [5]**	Experimental tumour-immune models using human NK/T cells, tumour-infiltrating lymphocytes and *Fusobacterium nucleatum* strains.	*Fusobacterium nucleatum*; Fap2-TIGIT interaction.	Showed that F. nucleatum Fap2 directly interacts with TIGIT and suppresses NK-cell and T-cell antitumor activity.	Provides an organism-specific mechanism by which a CRC-associated bacterium can contribute to local immune evasion.
**Rubinstein et al., 2013 [6]**	Experimental colorectal cancer cell and tumour models.	*Fusobacterium nucleatum*; FadA-E-cadherin/beta-catenin signalling.	Demonstrated FadA-mediated binding to E-cadherin and activation of beta-catenin signalling associated with colorectal carcinogenesis.	Links a CRC-associated bacterium directly to host epithelial signalling.
**Chung et al., 2018 [7]**	ApcMin mouse model colonised with enterotoxigenic *Bacteroides fragilis*, with epithelial and immune analyses.	Enterotoxigenic *Bacteroides fragilis*; B. fragilis toxin.	Showed that B. fragilis toxin initiates a pro-carcinogenic inflammatory cascade requiring epithelial IL-17R, NF-kappaB and STAT3 signalling.	Provides an organism-specific mechanism connecting microbial exposure to pro-carcinogenic epithelial and myeloid inflammation.
**Pleguezuelos-Manzano et al., 2020 [8]**	Human intestinal organoids exposed to pks-positive *Escherichia coli*, with comparison to 5,876 human cancer genomes.	pks-positive *Escherichia coli*; colibactin.	Repeated exposure produced a distinct mutational signature in organoids that was also detected in human cancer genomes, predominantly CRC.	Provides direct evidence that a microbial genotoxin can leave a durable mutational imprint relevant to colorectal carcinogenesis.
**Sinha et al., 2016 [9]**	Human faecal microbiome and metabolome study: 42 CRC cases and 89 matched controls.	*Fusobacterium*, *Porphyromonas*, Enterobacteriaceae, Actinobacteria and multiple faecal metabolites.	Observed stronger overall microbe-metabolite correlations in CRC; metabolites statistically mediated selected CRC associations with *Fusobacterium* and *Porphyromonas*.	Supports joint interpretation of microbial and metabolite signals rather than taxonomic abundance alone.
**Kim et al., 2020 [10]**	Human stool metabolomics with paired microbiota profiles: 102 advanced adenomas, 102 matched controls and 36 CRC cases.	Bioactive lipids, including polyunsaturated fatty acids, secondary bile acids and sphingolipids; microbiome-metabolome associations.	Identified adenoma-associated faecal metabolomic signatures and robust associations between microbial taxa and metabolomic features; many metabolite changes were directionally similar in CRC.	Provides metabolic context for early colorectal neoplasia and supports microbiome-metabolome coupling at the cohort level.
**Sun et al., 2024 [11]**	Four independent human cohorts totalling 1,251 participants: 422 CRC, 399 colorectal adenoma and 430 normal controls; experimental follow-up in CRC cells, organoids and mouse models.	Oleic acid, allocholic acid and other plasma/faecal metabolites.	Identified metabolites shifting across normal-adenoma-CRC states. Oleic acid was CRC-enriched and pro-tumorigenic, whereas allocholic acid was CRC-depleted and showed opposing effects.	Provides functional evidence that selected metabolites associated with colorectal neoplasia can directly influence CRC-relevant biology.
**Nogal et al., 2026 [12]**	Prospective long-term study of 354 women approximately 12 years after adenoma resection and 1:1-matched controls; faecal metabolomics in 184 matched pairs, with comparison to 14 CRC case–control studies.	Persistent microbiome alterations; sphingolipid-enriched metabolomic changes; adenoma-CRC shared microbial features.	The post-adenoma microbiome differed from controls and partially resembled CRC-associated alterations; shared microbes were also related to lifestyle factors.	Supports the possibility that some adenoma-concordant signals reflect durable or pre-existing host-microbial states rather than changes caused only by an active lesion.
**Dovrolis et al., 2024 [13]**	Pan-cancer analysis of TCGA tumour transcriptomes and cancer-cell-line expression data.	Microbial metabolite receptor genes, including HCAR1, HCAR3, serotonin-related and purinergic receptor systems.	Identified cancer-type-specific changes in microbial metabolite receptor gene expression; HCAR1 and HCAR3 were among the strongly upregulated receptors in colon and rectal adenocarcinoma.	Provides the host-side receptor framework used to organise microbial pathway signals in the present study. Bulk expression does not establish cell-type-specific receptor activity.
**Donohoe et al., 2012 [14]**	Experimental normal colonocyte and colorectal cancer cell models.	Butyrate and host metabolic state.	Showed that butyrate is oxidised as an energy substrate in normal colonocytes but accumulates and acts as an HDAC inhibitor in glycolytic cancer cells.	Demonstrates that the effect of the same microbial metabolite depends on host–cell metabolic context, cautioning against a unitary interpretation of SCFA-related signals.
**Lavoie et al., 2020 [15]**	ApcMin mouse models with global or dendritic-cell-specific Ffar2 disruption, with colitis/tumour experiments and host immune/barrier analyses.	FFAR2 signalling in dendritic cells.	Loss of FFAR2 increased colon tumourigenesis, impaired barrier integrity, increased tumor bacterial load and promoted exhausted/dying CD8 T cells and IL-27-producing dendritic-cell phenotypes.	Supports a functional role for host fatty-acid sensing in intestinal barrier, immune and tumour biology.
**Sivaprakasam et al., 2016 [16]**	Mouse intestinal carcinogenesis models, with supporting analysis of human colon cancer and matched adjacent tissue.	FFAR2/GPR43, dietary fibre and gut microbiota.	Ffar2-deficient mice were more susceptible to intestinal carcinogenesis, and dietary fibre suppressed carcinogenesis in an Ffar2-dependent manner while promoting a more beneficial microbial configuration.	Supports biological relevance of the SCFA-FFAR2 axis in intestinal carcinogenesis.

**He et al., 2025 [17]**	CRC cell, mouse tumour and human tumour-specimen analyses.	Lactate-HCAR1 signalling.	HCAR1 activation induced CCL2/CCL7 expression in colorectal tumour cells and promoted recruitment of immunosuppressive CCR2-positive PMN-MDSCs; Hcar1 loss reduced tumour burden in mice.	Provides direct functional evidence that host lactate sensing can reshape the CRC immune microenvironment.
**Teng et al., 2023 [18]**	Longitudinal locally advanced rectal cancer cohort with faecal microbiome and multi-omics profiling, plus cell and animal validation.	*Bacteroides vulgatus*-mediated nucleotide biosynthesis.	Microbial nucleotide biosynthesis was associated with resistance to neoadjuvant chemoradiotherapy; nucleoside supplementation or B. vulgatus gavage protected cancer cells from 5-fluorouracil or irradiation.	Provides functional evidence linking microbial nucleotide availability with treatment-response biology.
**Clos-Garcia et al., 2020 [25]**	Human controls, advanced adenoma and CRC; faecal UHPLC-MS metabolomics and 16S microbiome profiling (224 microbiome-profiled samples).	*Fusobacterium*, *Parvimonas*, *Staphylococcus*, Lachnospiraceae; cholesteryl esters and sphingolipids.	Reported CRC-associated microbial changes together with altered lipid metabolites and integrated microbiome-metabolome relationships.	Provides independent evidence that microbial and metabolomic remodelling co-occur in colorectal neoplasia.
**Dillard et al., 2021 [26]**	Four CRC cell lines (HT29, LS513, LS174T and HCT116) compared with normal-like colonic epithelial cells, with 2D/3D expression and functional assays.	Extracellular ATP/adenosine signalling; P2Y/P2X/ADORA receptors; CD39 and CD73 ectonucleotidases.	Characterised purinergic receptor expression and cellular responses to ATP and adenosine. CD39 was poorly or not expressed in the CRC cell-line panel, whereas CD73/NT5E was slightly downregulated relative to normal-like cells.	Shows that local purinergic effects depend on receptor expression and host extracellular nucleotide-processing machinery rather than microbial nucleotide pathways alone.
**Sun et al., 2020 [27]**	Human cohort: 46 sporadic CRC, 33 colon adenoma and 38 healthy controls, with faecal tryptophan metabolites, 16S microbiota and host barrier/tissue measurements.	Tryptophan, kynurenine and microbial indole metabolism.	CRC showed increased faecal kynurenine-related measures and a reduced indoles-to-tryptophan ratio, with correlations between indole-related measures, microbiota and intestinal barrier markers.	Supports remodelling of tryptophan metabolism in CRC while illustrating that microbial indole and host kynurenine branches are biologically distinct.
**Du et al., 2022 [28]**	Human faecal microbiome and metabolome study: 30 CRC patients and 36 controls.	Tryptophan/indole-related metabolism, fatty acids, monosaccharides and CRC-associated microbial taxa.	Reported increased faecal tryptophan, lower indoleacrylic acid, and correlations linking tryptophan/indole-related metabolites with *Ruminococcus bicirculans* and *Fusobacterium*.	Supports altered microbiome-metabolome coupling within the tryptophan/indole branch in CRC, without establishing the direction of host receptor signalling.
**Vyhlidalova et al., 2020 [29]**	In vitro receptor-binding, reporter-gene and intestinal/liver cell assays.	Gut microbial tryptophan catabolites and aryl hydrocarbon receptor (AHR).	Demonstrated that multiple microbial tryptophan catabolites can act as AHR ligands/agonists, with substantial differences in potency, efficacy and antagonist behaviour.	Provides receptor-level evidence that microbial tryptophan-derived metabolites can directly engage host AHR signalling.
